# Transcranial Direct Current Stimulation to Improve the Dysfunction of Descending Pain Modulatory System Related to Opioids in Chronic Non-cancer Pain: An Integrative Review of Neurobiology and Meta-Analysis

**DOI:** 10.3389/fnins.2019.01218

**Published:** 2019-11-18

**Authors:** Maxciel Zortea, Leticia Ramalho, Rael Lopes Alves, Camila Fernanda da Silveira Alves, Gilberto Braulio, Iraci Lucena da Silva Torres, Felipe Fregni, Wolnei Caumo

**Affiliations:** ^1^Post-graduation Program in Medicine: Medical Sciences, Universidade Federal do Rio Grande Do Sul (UFRGS), Porto Alegre, Brazil; ^2^Laboratory of Pain & Neuromodulation, Hospital de Clínicas de Porto Alegre (HCPA), Porto Alegre, Brazil; ^3^Service of Anesthesia and Perioperative Medicine, Hospital de Clínicas de Porto Alegre (HCPA), Porto Alegre, Brazil; ^4^Department of Pharmacology, Institute of Health Sciences (ICBS), Universidade Federal do Rio Grande Do Sul (UFRGS), Porto Alegre, Brazil; ^5^Pharmacology of Pain and Neuromodulation: Pre-clinical Investigations Research Group, Universidade Federal do Rio Grande Do Sul (UFRGS), Porto Alegre, Brazil; ^6^Neuromodulation Center, Spaulding Rehabilitation Hospital, Harvard Medical School, Boston, MA, United States; ^7^Pain Treatment and Palliative Medicine Service, Hospital de Clínicas de Porto Alegre (HCPA), Porto Alegre, Brazil

**Keywords:** tDCS, hyperalgesia, opioid, pain, descending pain inhibitory system

## Abstract

**Background:** Opioid long-term therapy can produce tolerance, opioid-induced hyperalgesia (OIH), and it induces dysfunction in pain descending pain inhibitory system (DPIS).

**Objectives:** This integrative review with meta-analysis aimed: (i) To discuss the potential mechanisms involved in analgesic tolerance and opioid-induced hyperalgesia (OIH). (ii) To examine how the opioid can affect the function of DPIS. (ii) To show evidence about the tDCS as an approach to treat acute and chronic pain. (iii) To discuss the effect of tDCS on DPIS and how it can counter-regulate the OIH. (iv) To draw perspectives for the future about the tDCS effects as an approach to improve the dysfunction in the DPIS in chronic non-cancer pain.

**Methods:** Relevant published randomized clinical trials (RCT) comparing active (irrespective of the stimulation protocol) to sham tDCS for treating chronic non-cancer pain were identified, and risk of bias was assessed. We searched trials in PubMed, EMBASE and Cochrane trials databases. tDCS protocols accepted were application in areas of the primary motor cortex (M1), dorsolateral prefrontal cortex (DLPFC), or occipital area.

**Results:** Fifty-nine studies were fully reviewed, and 24 with moderate to the high-quality methodology were included. tDCS improved chronic pain with a moderate effect size [pooled standardized mean difference; −0.66; 95% confidence interval (CI) −0.91 to −0.41]. On average, active protocols led to 27.26% less pain at the end of treatment compared to sham [95% CI; 15.89–32.90%]. Protocol varied in terms of anodal or cathodal stimulation, areas of stimulation (M1 and DLPFC the most common), number of sessions (from 5 to 20) and current intensity (from 1 to 2 mA). The time of application was 20 min in 92% of protocols.

**Conclusion:** In comparison with sham stimulation, tDCS demonstrated a superior effect in reducing chronic pain conditions. They give perspectives that the top-down neuromodulator effects of tDCS are a promising approach to improve management in refractory chronic not-cancer related pain and to enhance dysfunctional neuronal circuitries involved in the DPIS and other pain dimensions and improve pain control with a therapeutic opioid-free. However, further studies are needed to determine individualized protocols according to a biopsychosocial perspective.

## Introduction

A recent national survey estimates that 3–4% of Americans adults have been receiving long-term opioid therapy (Dowell et al., 2016), and it estimated that almost 2 million meet the DSM-IV criteria for dependence or abuse (Florence et al., 2016). Chronic pain syndromes, defined as recurrent or persistent pain lasting 3 months, can be related to structural changes such as a decrease in neocortex gray matter (Geha et al., 2009). Even if research signs of progress and new targets appear for treating acute and chronic pain, opioids still representing the gold standard analgesics. However, opioid treatments induce several side effects, among them, are the analgesic tolerance and opioid-induced hyperalgesia (OIH). These phenomena are closely related to the dysfunction of the descending pain inhibitory system (DPIS). Hence to comprehend the relevance of new therapeutic approaches in this field, it is essential an integrated view of their potential properties to block processes involved in the neurobiology of tolerance and OIH. Thereby, we conduct the reader to a brainstorm of this question to integrate concepts related to pain, opioids effects as neuromodulators (analgesia, tolerance, and OIH and dysfunction of descending pain inhibitory system) and the impact of transcranial neuromodulatory techniques. Also, we present some essential technical aspects of how to use of tDCS, evidence about their effect on pain and future perspectives.

### An Integrative View of Chronic Pain Opioid Use and Transcranial Neuromodulation

Chronic pain is maladaptive response related to a reduction in the neurogenesis at the hippocampus (Lanz et al., 2011) and a decreased volume of the ventromedial prefrontal cortex (VMPFC) (Abdallah and Geha, 2017). Other changes found in chronic pain were a gray matter density decrease in the cerebral cortex, specifically in areas such as cingulate, insular, prefrontal and dorsolateral, somatosensory, thalamus, motor cortex, and brainstem (Kuchinad et al., 2007). These changes include neurodegeneration related to the severity of pain (Lefaucheur et al., 2006, 2011; May, 2008). Aligned with these neuroimage findings, neurophysiological measures by transcranial magnetic stimulation (TMS) indicate a dysfunction in the cortical excitability measures and the corticospinal pathways (Niddam and Hsieh, 2009). Such changes include increased motor evoked potential (MT), cortical silent period reduction (CSP) and short intracortical inhibition decrease (ICI), which suggest a disruption of cortical inhibition mechanisms (Gussew et al., 2011; Huang et al., 2017; Cardinal et al., 2019). According to Lefaucheur et al. (2006), the lack of inhibitory mechanisms contributes to increasing the cortical excitability and indicates an imbalance in GABAergic and glutamatergic systems (Mhalla et al., 2010). These dysfunctions in pain processing also include altered intrinsic brain connectivity. In chronic pelvic pain related to endometriosis, it was found greater connectivity between the anterior insula region with the medial prefrontal cortex (mPFC). The connectivity level was correlated positively with the severity of pain, anxiety, and depression (As-Sanie et al., 2016). Similar results were found in fibromyalgia (Napadow et al., 2010, 2012), and chronic low back pain (Loggia et al., 2013).

Likewise, in persons addicted to opioids, a decreased functional connectivity in brain regions such as the nucleus accumbens, anterior insula, and amygdala subdivisions has been involved in the regulation of affect, impulse control, reward, and motivational functions. Besides, more prolonged exposure to opioid prescription is correlated with a bilateral volumetric loss in the amygdala compared to controls (Upadhyay et al., 2010). Although some of these changes likely seem reversible after pain treatment (e.g., gray matter volume loss; Maarrawi et al., 2013) and after treatment with pregabalin (Kim J. et al., 2013) and acupuncture (Napadow et al., 2012), both treatments reduced the connectivity in these regions of the default mode network (DMN) related to pain processing. These set of symptoms reflect changes responsible for the shift of the sensory system physiological to pathological pain hypersensitivity, which comprises mechanisms of central sensitization syndrome (CSS) (Yunus, 2015).

The International Association for the Study of Pain (IASP) defines “central sensitization” broadly as the “increased responsiveness in the pain pathways neurons to their normal or subthreshold afferent input.” This phenomenon is a consequence of perturbation of the nociceptive system with the peripheral or central sensitization, and it is involved in the hyperalgesia (Brietzke et al., 2019). In chronic pain, the CCS is linked to the cognitive depressed mood, fatigue, and catastrophizing thoughts and disability due to pain (Caumo et al., 2017; Brietzke et al., 2019). This increased excitability and reduced inhibition in the systems of pain processing increase the gain of incoming sensory information, resulting in exaggerated pain, secondary hyperalgesia, allodynia, and temporal summation (Staud et al., 2001; Price et al., 2002).

Although we have witnessed a leap forward in our comprehension of the mechanistic underpinnings of pain, and the potential treatment targets have grown, it is still persistent a gap between pain research and the pain management in the clinical setting, which remains to be a challenge. Among drugs currently used to treat chronic pain, opioids are frequently prescribed. In the US, ~3–4% of the adult population received long-term opioid therapy. However, this prescription is not supported by evidence, because the efficacy of opioids in randomized clinical trials lasts primarily 12 weeks or less (Dowell et al., 2016). Nonopioids drugs currently used include selective norepinephrine or serotonin reuptake inhibitors (SNRIs and SSRIs), membrane stabilizers (e.g., anticonvulsants), acetaminophen, non-steroidal anti-inflammatory drugs (NSAIDs), etc. (Nicol et al., 2017). Even though these drugs are FDA-approved, according to the criteria of IMMPACT consensus statement (Gewandter et al., 2015), which defines an effect on pain reduction of considerable magnitude of at least a 50% (alternatively 30% or moderate pain relief), and the effect sizes for many of these treatments are small (Nicol et al., 2017). Another aspect to consider is their side-effects, particularly with opioids, such as sedation, nausea, and their reduced long-term efficacy due to receptor downregulation.

According to the extensive literature in non-cancer chronic pain, the prescription of opioid drugs should consider a proper balance between the benefits and the risk for addiction. Such factors associated with opiate dependence, include psychosocial problems (e.g., childhood neglect, abuse, or trauma), mental disorders (e.g., depression and anxiety) and to have a history of another dependence (Busse et al., 2017). The reliance of substance comprises tolerance, defined by either a markedly increased amounts of the abuse substance to the desired effect and a diminished effect with continued use and withdrawal symptoms (Busse et al., 2017). In addition to the dependence, the opioids can elicit an unexpected increase in pain sensitivity. This phenomenon is known as hyperalgesia induced by opioids (HIO). Its mechanism remains still obscure, even though abnormal functional features have been identified, mainly by imaging and neurophysiological measures. Although the OIH and the tolerance may share some neurochemical mechanisms, at the clinical setting cannot define where one ends and initiates the other or if they are a continuum of the same phenomenon. The OIH and dependence, both can be involved in chronic pain refractory, and they can constitute a mechanism underpinning the inadequate pain control, either in opioid use or in addicts to opiate.

Thus, therapeutic approaches that alter the membrane potential and could change the dysfunctional plasticity within pain circuits, they may also affect the nuclei in the thalamus and subthalamic regions (Strafella et al., 2004; Lang et al., 2005; Moreno-duarte et al., 2014). Taking this into account is plausible that the repetitive transcranial magnetic stimulation (rTMS) and transcranial direct current stimulation (tDCS) may be promising alternatives for this context. Although the FDA approved the TMS for the treatment of major depressive disorder symptoms treatment-resistant after use of two or three antidepressant medicines with lack of adequate response (Nemeroff, 2007) and the rTMS was approved for chronic neuropathic pain in Europe (Lefaucheur et al., 2006; André-Obadia et al., 2014), it has not been approved for chronic pain treatment yet. At present, the device to apply TMS is relatively expensive and need to be done under direct supervision in medical centers. tDCS, on the other hand, is a cheaper and easier-to-use type of stimulation technique. TMS pulse can generate currents capable of depolarizing neurons until it reaches the threshold for firing action potentials, whereas tDCS modulates the resting membrane potential and require less sophisticated equipment than TMS. The tDCS requires two electrodes, cathode, and anode, arranged in different positions to create a direct current flow of low intensity (1 or 2 mA) that targets a specific region of the cerebral cortex. However, it is essential to consider that its effects, as well as the effect of the pharmacological treatment, can be modified by genetic polymorphisms of BDNF and monoamines [e.g., monoamine oxidase (MAO), and to catechol-O-methyltransferase (COMT)] and several other factors such as sex, age, central nervous disease, medicines with effect in the central nervous system, etc. Also, tDCS effects are likely to be neuroplasticity state dependent, since previous study found that serum BDNF predicts the impact of tDCS on behavioral measures in chronic pain conditions (Souza et al., 2018).

This integrative review with meta-analysis has the following aims: (i) To discuss the potential mechanisms involved in analgesic tolerance and opioid-induced hyperalgesia (OIH). (ii) To examine how the opioid can affect the function of descending pain inhibitory system (DPIS). (ii) To show evidence about the tDCS as an approach to treat chronic pain. (iii) To discuss the effect of tDCS on DPMS and how its impact can counter-regulate the OIH. (iv) To draw perspectives for the future about the tDCS effects as an approach to improve the dysfunction in the DPIS in chronic non-cancer pain.

### Opioids Effects as Neuromodulators: Analgesia, Tolerance, and OIH and Dysfunction of Descending Pain Inhibitory System

#### Analgesia Induced by Opioids

**Figure 1:**
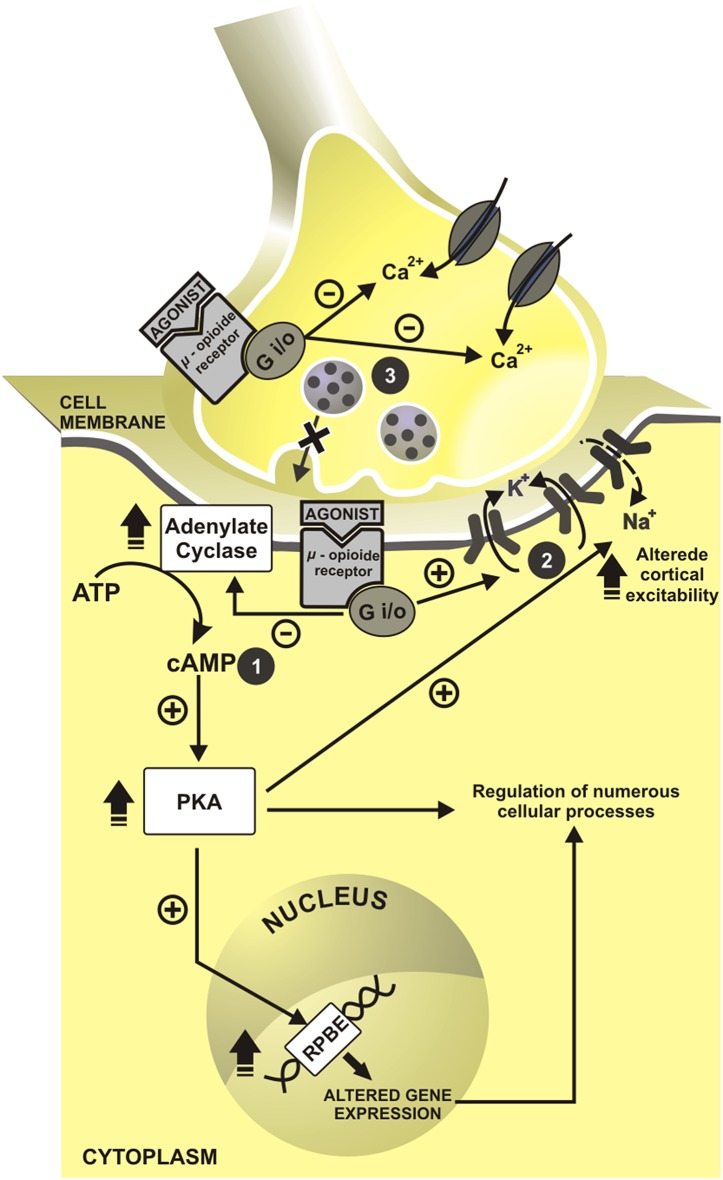
Cell mechanisms of opioids action. In presynaptic neuron have inhibition of intracellular calcium influx and impairment in neurotransmitters release. In a postsynaptic neuron, cascade events induce to hyperpolarization state. cAMP, cyclic adenosine monophosphate; RPBE, response of cAMP to the protein binding element; Ca^2+^, calcium; K^+^, potassium; Na^+^, sodium; +, excitatory; –, inhibitory. 1. Inhibition of adenylate cyclase reduces the cyclic adenosine monophosphate (cAMP); and second messengers. 2. With this, cAMP reduction allow the openness of the potassium channels and promote the postsynaptic cell hyperpolarization. 3. The concomitant activation of presynaptic opioid receptors of C and Aδ fiber inhibits indirectly calcium influx, which decreases cAMP levels and blocks neurotransmitters release—glutamate, substance P and calcitonin gene-related peptide (CGRP).

More than 200 years ago, the German Friedrich Sertürner isolated an alkaloid Crystal from opium, extracted from poppy, and called it morphine. After the determination of morphine structure in 1920, the synthesis of new opioids compounds derived from morphine and based on its chemical structure has begun (Huxtable and Schwarz, 2001). In 1939, meperidine emerged as the first entirely synthetic opioid. Fentanyl, another opioid, was available since 1960. Between 1974 and 1976, were developed some fentanyl analogs: carfentanil (1974), sufentanil (1974), lofentanil (1975), alfentanil (1976) (Janssen, 1982) and in the early 90's remifentanil was available for clinical use. Opioids bind to receptors in the central nervous system (CNS) μ (MOR), δ (DOR), κ (KOR). Another type of receptor is ORL-1, mostly located in the solitary tract nucleus, periaqueductal gray matter, frontal cortex, thalamus, and central gelatinous substance of the spinal cord (Butour et al., 1998). These opioids receptors are opioid conjoining to inhibitory G—(iG)-protein (Waldhoer et al., 2004), and they can be agonist-antagonists. The clinical effects include analgesia and sedation, which are produced by a reduction to intracellular calcium influx and to impair the quantity of the neurotransmitters in the cleft synapsis. This cascade of events that lead to hyperpolarization of membranes is presented in Figure 1.

#### Tolerance to Opioids Analgesic Effects

**Figure 2:**
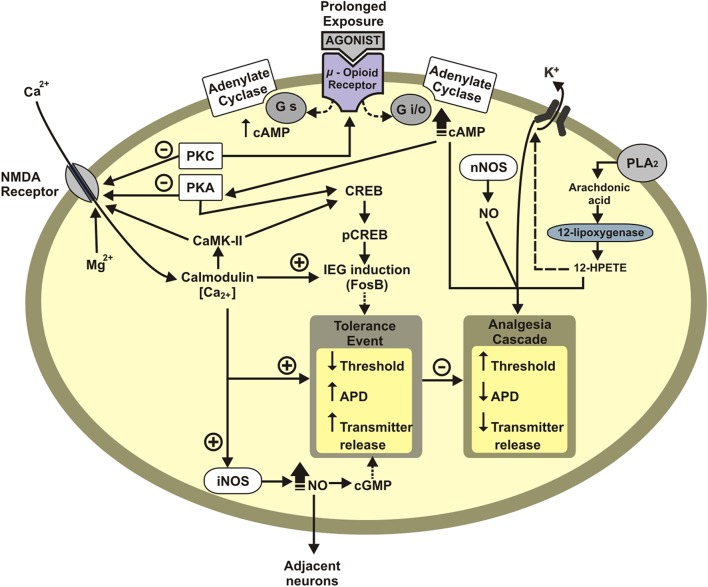
Schematic representation of neuronal mechanisms underlying opioid tolerance development. Tolerance event: Decreases membrane potential threshold, increases the action-potential duration (APD), and increases neurotransmitter release. APD, action-potential duration; IEG, immediate early genes (c-Fos, FosB); PKA, protein Kinase A; CREB, cAMP response element-binding protein; pCREB, phosphorylated CREB protein; Gi/o, inhibitory G protein; Gs, excitatory G protein; CaMK-II, calcium/calmodulin dependent protein kinase II; PLA2, phospholipase A2; NO, nitric oxide; nNOS, neuronal nitric oxide synthase; HPETE, hydroperoxyeicosatetraenoic acid; +, excitatory; –, inhibitory.

Tolerance to opioid analgesia involves the desensitization of Mu opioid receptor (MOR) (see Dang and Christie, 2012; Williams et al., 2013). The desensitization corresponds to a loss of effectiveness to agonist by repeated exposure (Dang and Christie, 2012; Williams et al., 2013). The homologous desensitization, i.e., the activation of a receptor type provokes its desensitization (i.e., MOR activation desensitizes MOR), and heterologous desensitization when the activation of another receptor desensitizes MOR, such as the desensitization described between MOR and chemokine receptors (CxCRs) (see Parsadaniantz et al., 2015). For instance, the desensitization of MOR by the interaction between MOR-CxCr4 and MOR-Cx3Cr1 in the periaqueductal gray matter (PAG) (Heinisch et al., 2011). Neurons treated *in vitro* with the respective CxCr4 or Cx3Cr1 agonists displayed decreased morphine-induced electrophysiological activity. And, intraspinal CXCL12 administration diminished morphine analgesia, while CXCR4 antagonist potentiated morphine analgesia (Rivat et al., 2014). At a molecular view, CxCRs activate protein kinase C (PKC), which phosphorylates the intracytoplasmic tail of MOR (see Williams et al., 2013). MOR phosphorylation uncouplers MOR from Gi [inhibitory protein-conjoined receptor (GPCR)]. Then, G protein receptor kinase (GRK) and arrestin are recruited to MOR, leading to MOR internalization and the result this cascade is the analgesic tolerance (see Williams et al., 2013). CxCR activation can also lead to activation of signal-regulated kinase (ERK) in the pathway that decreases nociceptive thresholds and hence induces hyperalgesia (see Parsadaniantz et al., 2015). The mechanisms involved in the tolerance are presented in Figure 2.

The dependence motivating effects of abuse drugs occur through the activation of the mesocorticolimbic dopaminergic pathway, a central system that mediates the effects of reinforcement (Uhl et al., 2019). The primary circuit of this system comprises dopaminergic neurons in the ventral tegmental area, which has connections with prefrontal cortex and accumbens nucleons, while the emotional aspects of dependence memories are associated with amygdala, hippocampus, and hypothalamus. The amygdala is involved in the conditioned response and gives the emotional value to perception after drug use. Also, the amygdala participates in emotional memory, providing a positive or negative value of new information, whereas the hypothalamus receives the afferents of the nucleus accumbens to trigger an autonomic and neuroendocrine response (Uhl et al., 2019).

The most common strategy to face opioid dependence is pharmacological, which comprises methadone, used to treat the Opioid Use Disorders (OUD). The buprenorphine, naltrexone are equally efficacious in non-injection opioid analgesia users (Potter et al., 2013). Despite methadone being a mu opioid receptor agonist and one an NMDAr antagonist, OIH can still develop (Compton et al., 2012). A high number of patients with OUDs reported persisted with chronic pain, even after conversion to methadone (Compton et al., 2012; Dennis et al., 2015). Although behavioral and pharmacological therapies have been used to treat abstinence in alcohol and drug abuse, relapse rates continue higher than 60% (HHS and Office of the Surgeon General, 2016). This suggests a need for further research to develop more effective treatments.

#### Hyperalgesia Induced by Opioids

The HIO seem to depend on complex interactions between the various cell types that make up the central nervous system, such as the activation of opioid receptors in neurons, astrocytes, and microglia, associated with the release of pro-inflammatory cytokines, events crucial for the understanding of the mechanism of opioid-induced hyperalgesia. In neurons, activation of opioid receptors may decrease glutamate reuptake in the synaptic cleft, in addition to increasing NMDA receptor activity. Still in neurons, the morphine-induced astrocyte BDNF release, which causes a decrease in Cl- transporters of type KCC2, reversing the concentration gradient of this ion. In neuropathic and chronic inflammatory pain conditions, GABAergic inhibitory control is decreased, leading to increased excitation and central sensitization (Malcangio, 2018).

According to Ferrini et al. (2013), rats treated with morphine for seven days altered Cl- homeostasis (together with a downregulation of the K+-Cl– co-transporter KCC2) homeostasis in spinal lamina l neurons. Subsequently, it was observed a reversal potential for GABAa currents, making neurons become more depolarized. When exogenous GABA was applied, they saw GABA that it is evoking a biphasic response, initially with an outward flow, which was followed by a shift to an inward current. This shift was not found in the case of saline-treated rats. Therefore, it is plausible to conclude an excitatory effect had taken place, which was associated with hyperalgesia. When researchers blocked BDNF-TrkB signaling, it was observed the preservation of Cl– homeostasis and a reduction in hyperalgesia. In astrocytes, chronic-activated opioid receptors may promote the release of pro-inflammatory cytokines, such as tumor necrosis factor alpha (TNFα), interleukin-1β (IL-1β), IL-6, IL-10, monocyte chemotactic protein 1 (CCL2) and chemokine CXC 1 (CXCL1). Besides, morphine can inhibit astrocyte glutamate transporters, promoting an increase in the extracellular concentration of this neurotransmitter (Roeckel et al., 2016).

On the other hand, opioid receptors activated in microglia leads to a rise in the expression of purinergic ionotropic receptors P2X4 and P2X7, as well as Toll 4 type receptors (TLR4) (Hutchinson et al., 2011; Grace et al., 2014) further increasing the release of pro-inflammatory cytokines, as well as ATP and nitric oxide (Zhou et al., 2010). In turn, the cytokines released from astrocytes and microglia will activate their respective receptors located in nociceptive neurons, causing desensitization of the opioid receptors, which will cause a decrease in the analgesic effect (Roeckel et al., 2016). The acute and chronic morphine administration activate cytokines released by astrocytes. Acute morphine exposure over satellite glial cells in the dorsal root ganglia (DRG) leads to upregulation of inflammatory cytokine interleukin 1-beta (IL-1β). Likewise, chronic morphine administration demonstrates upregulation of inflammatory cytokine (IL-1β) in spinal astrocyte (Berta et al., 2012). About chronic use of morphine, microglia cells also release proinflammatory cytokines (Merighi et al., 2013). The opioid desensitization receptor occurs by distinct mechanisms of desensitization potential between opioid receptors and inflammatory substances that participate in the G-protein-coupled systems (Szabo et al., 2002). Other neurons and glia systems (calcium, MAP kinases pathway, and nuclear factor-κB) also may be acting in this different desensitization (Haddad, 2002).

**Figure 3:**
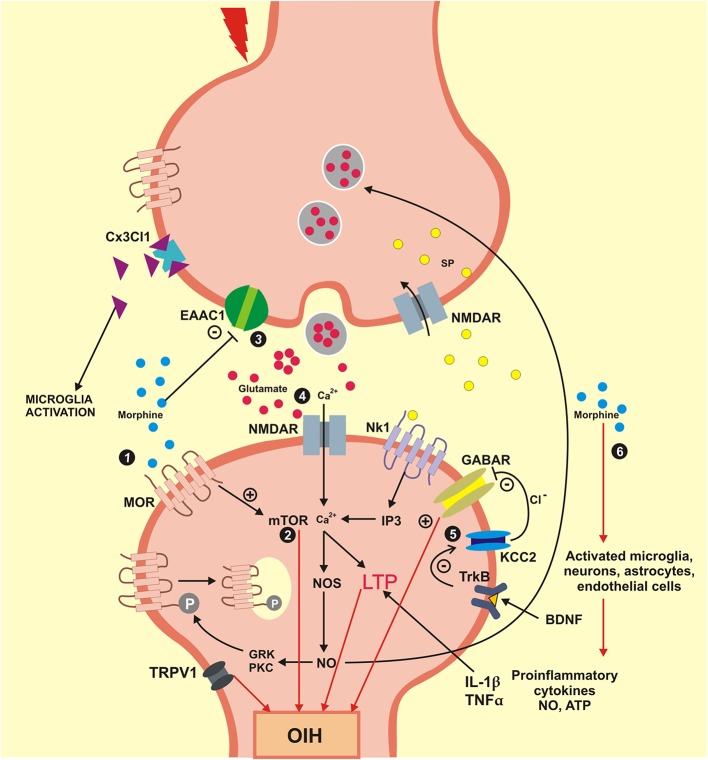
Mechanisms engaged in the formation and maintenance of OIH. OIH, Hyperalgesia induced by opioids; BDNF, brain derived neurotrophic factor; Cx3Cl1, chemokine Cx3Cl1; EAAC1, glutamate transporter EAAC1; GABAR, gamma aminobutyric acid receptor; GRK, G protein coupled receptor kinase; IL1β, interleukin beta; IP3, inositol triphosphate; KCC2, K+/Cl cotransporter 2; LTP, long-term potentiation; MOR, mu-opioid receptor; mTOR, mammalian target of rapamycin; NO, nitric oxide; NOS, nitric oxide synthase; NK1, neurokinin 1 receptor; NMDAR, N-methyl-D-aspartic acid receptor; PKC, protein kinase C; SP, substance P; TNFα, tumor necrosis factor alpha; TrkB, tyrosine kinase B; TRPV1, transient receptor potential vanilloid 1; Ca^2+^, calcium; Cl^−^, chlorine; +, excitatory; –, inhibitory. (1) Morphine activates the neuronal MOR and generates an intracellular process that culminates in the OIH; (2) Signaling mTOR is activated by morphine engaged in neuroexcitation that lead to OIH; (3) The synaptic concentration of glutamate is elevated when the glutamate transporter EEAC1 are blocked by morphine. (4) Glutamate coupling in your NMDA receptors activates the calcium influx inducing the LTP that lead an OIH. (5) The BDNF receptor TrkB reduces the action of the cotransporter KCC2 evolved in ions chlorine homeostasis that modifies the inhibitory function of GABA for an excitatory function that which promote OIH. (6) Morphine also activates the glial cells and neurons that generate pro-inflammatory cytokines (IL1β and TNFα) that induce LTP and lead to OIH.

Also, activation of cytokine receptors in neurons causes an increase in the expression of excitatory NMDA-like glutamate receptors leading to increased sensitivity to pain (Roeckel et al., 2016). Hence, these neuronal processes contribute to activation of neuroexcitatory mechanisms involved in long-term potentiation (LTP) and descending pain facilitation (Chu et al., 2011; Lee et al., 2011). Together, these cellular and molecular mechanisms lead to the sensitization of neurons and contribute to the development of hyperalgesia induced by morphine and its derivatives. The OIH is pronociceptive changes that occur in combined with catecholamine release, and other factors have been linked with withdrawal-induced hyperalgesia, or on long-term opioid therapy for chronic pain (Bie et al., 2003). For clinical purposes, these two phenomena are difficult to handle, since the development of analgesic tolerance will lead to an increase in the opiate dosage, which in turn will induce an enhancement of OIH. The neuronal pathways involved in OIH, such as activation of neuronal MOR and the regulation of intracellular mechanisms involved in OIH are presented in Figure 3.

### Impact of Genetic Polymorphisms in the Pain Modulatory System and Treatment Effect

The susceptibility to opioid physical dependence, tolerance, and opioid-induced hyperalgesia (OIH) display a significant degree of heritability (>60%) (Kest et al., 2002; Wilson, 2003; Liang et al., 2006a,b). In humans, studies with twin and families estimated that 40–60% of opioid dependence is driven by genetic factors (Yuferov et al., 2010). Although few causal genetic variants have been identified (Gelernter et al., 2014), the impact of these genetic aspects on the opioid dependence need to be determined. Serum BDNF levels were found to be significantly higher in Val/Val carriers than in Met/ Val or Met/Met in heroin users (Roviš et al., 2018). Besides, some prefrontal cortical areas are involved in the inhibition of nociception (Petrovic et al., 2002). The descending pain inhibitory pathway is modulated by the central catecholaminergic systems (Basbaum and Fields, 1979). The functional polymorphism Val158Met (rs4680) of the Catechol-O-methyltransferase (COMT) gene, which regulates the metabolism of dopamine and noradrenaline can be related to higher pain sensitivity. The Val158Met variant of the substitution of the amino acid valine (Val) for methionine (Met) at codon 158 is related to a breakdown of dopamine and noradrenaline up to four times for the valine allele compared to methionine. Hence, they have available lower levels of dopamine/noradrenaline in the synaptic cleft (Lotta et al., 1995). In the same way, the Val158Met polymorphism of COMT can influence the impact of the tDCS on pain and depression. The polymorphisms of BDNF has been pointed out as a factor that influences the neuromodulatory effect of pharmacological (e.g., morphine, antidepressants, etc.) and non-pharmacological therapies (tDCS, TMS, etc.).

The Val66Met (rs6265) polymorphism of the BDNF gene resulted in the substitution of an amino acid valine to methionine in the pro-BDNF peptide. Then, three genotypes are possible Val/Val, Val/Met, or Met/Met, respectively (Mowla et al., 2001; Hariri et al., 2003). This polymorphism has been extensively studied and may affect the secretion of BDNF (Egan et al., 2003), regulate cell survival, growth, and modulate synaptic changes (Mowla et al., 2001; Hariri et al., 2003; Frielingsdorf et al., 2010). Met carriers were associated with the reduction of volume in the hippocampus (Pezawas et al., 2004; Reiser et al., 2007), and dorsolateral prefrontal cortex. Val66Met polymorphism is associated with cortical maturation in children and adolescents with and without psychiatric disorders (de Araujo et al., 2018). Met carriers were also associated with an increased risk for anxiety trait (Arias et al., 2012), suicide behavior in Asian and Caucasian population (González-Castro et al., 2017) and alcohol dependence (Matsushita et al., 2004). On the other hand, in Han Chinese, higher Val frequency was found in heroin users. However, Val allele carriers had a later onset of heroin abuse compared to Met allele carriers (Cheng et al., 2005). In addition, researchers found a significant association between BDNF Val66Met and worse treatment outcomes among Met carriers (Heinzerling et al., 2012). Besides, Met carriers reported more time- and cost-intensive heroin-seeking behavior than did carriers of the Val/Val (Greenwald et al., 2013).

BDNF is a neurotrophin that modulates long-term potentiation (LTP), and it has a central role in sensitization of pain pathways at the spinal level. In the presence of inflammation, the BDNF is upregulated in the lifetime of spinal cord injury it promotes a process of adaptive plasticity and functional recovery. Although the effect of BDNF is multifaceted, at the medullary level it is associated (i) expression of TrkB receptor and downstream kinases, (ii) modifies GABAergic transmission by altering the appearance of chloride channels, and (iii) activation of cells of astroglia. BDNF potentiate the metaplastic changes at the medullary level that are essential to spinal function, especially after spinal cord injury (Garraway and Huie, 2016). Although the findings are still incipient and not conclusive, BDNF levels seem to be related to anodal tDCS over the M1 in the body pain associated to pegylated-interferon (Brietzke et al., 2016) and TMS (Dall'Agnol et al., 2014). Another system involved in the pain processing and perhaps in the effect of treatment is the monoaminergic system, in which the COMT enzyme plays a critical role in the degradation of catecholamines, such as dopamine, where it transfers a methyl group of S-adenosylmethionine to the 3-hydroxy group of catechol (Axelrod and Tomchick, 1958). A functional polymorphism at position 108/158 of COMT causes a valine amino acid exchange for methionine (Val 108/158 Met) affects the thermal stability of the enzyme as well as its activity. The Met allele results in a more thermolabile and less active COMT phenotype (Lachman, 1996; Chen et al., 2004). The influence of serotonergic 5-HT1A receptor promoter region polymorphism predicted the outcome of the rTMS effect on depressive symptoms (Malaguti et al., 2011). When tDCS was applied to the DLPFC it produced a more significant reduction of auditory hallucinations in homozygous for Val allele of the COMT polymorphism compared to carriers of Met allele in schizophrenia (Shivakumar et al., 2015). In healthy subjects, two reports demonstrated a specific interaction of COMT polymorphism with both anodic and cathodic tDCS during executive functioning using a Go/No-Go task (Plewnia et al., 2013; Nieratschker et al., 2015). This task address different aspects of executive functioning: sustained attention, inhibition of response, and set change abilities (Langenecker et al., 2007). In both experiments, the anodal tDCS (1 mA) was applied together with a cognitive task over the DLPFC. In the first study, the effect of anodal tDCS was observed according to Val158Met polymorphism of the COMT. Specifically, it was found impaired by the set-displacement abilities, which in homozygous for Met allele indicates a deterioration of cognitive flexibility but not in carriers of Val allele. In short, COMT Val158Met polymorphism shown to shape the tDCS effects on executive functions. However, the number of studies examining this interaction is still small. Future studies are needed to give extent data of the impact of genetic polymorphisms on the variability of tDCS effects and to elucidate if they have an essential effect that would help to define individualized protocols in a translational research model. Besides, is necessary to investigate if the therapeutic effect of tDCS may interfere on the central mechanisms of pain and, if it could be an alternative or complementary therapy to standard treatment options (e.g., opioids, antidepressants, anticonvulsants).

### Dysfunction of Descending Pain Inhibitory System

As mentioned above, the disruption in the balance between excitatory and inhibitory neurobiological systems in opioids tolerance and OIH can be linked to the dysfunction in the DPIS. Although the somatotopic organization of DPIS influence is quite diffuse, the pain inhibitory (DPI) pathways originate in or relay through many brainstem nuclei namely the midbrain periaqueductal gray (PAG) and the rostral ventromedial medulla (RVM). The PAG-RVM system converges from other pain modulatory areas thalamic, hypothalamic, and telencephalic (cortical and subcortical) structures suppress pain through descending projections to the spinal dorsal horn (Pertovaara and Almeida, 2006). The descending circuitries involved in pain inhibition have provided feedback control of nociceptive signals at the spinal cord level (Pertovaara and Almeida, 2006). The PAG neurons project to the raphe neurons, where do DPIS effect is mediated by monoamines, peptides, and amino acids, and by several different types of neurophysiological mechanisms acting on central terminals of primary afferent nociceptive nerve fibers, spinal interneurons, and spinal projection neurons. The chronic pain may result in disorders of neurotransmitter systems, which potentially lead to a decrease of DPIS function or an increase of descending facilitation. The role of DPIS may be enhanced by some centrally acting drugs (e.g., drugs acting on the monoaminergic system, such as antidepressants with dual or tricycle effect) (Pertovaara and Almeida, 2006). The DPIS can be modulated either in a “top-down” manner, using approaches to stimulate brain areas involved in descending inhibitory controls [e.g., behavioral therapy, tDCS, transcranial magnetic stimulation (TMS), etc.], or in a “bottom up” activation with peripheral nerve stimulation (e.g., acupuncture, electroacupuncture, etc.). Hence a better comprehension of the relationship between the dysfunction in the DPIS induced by opioids may contribute to finding factors involved in the variability in pain sensitivity. Thereby, research can give support for clinicians to understand factors that perpetuate opioid use and barriers to detoxification (Volkow and McLellan, 2016). Aligned with this perspective the tDCS and other neuromodulatory approaches can help in the management of pain with lower risk of producing addiction, while it could reduce the suffering when starting opioids dose reduction protocols (Rieb et al., 2016).

**Figure 4:**
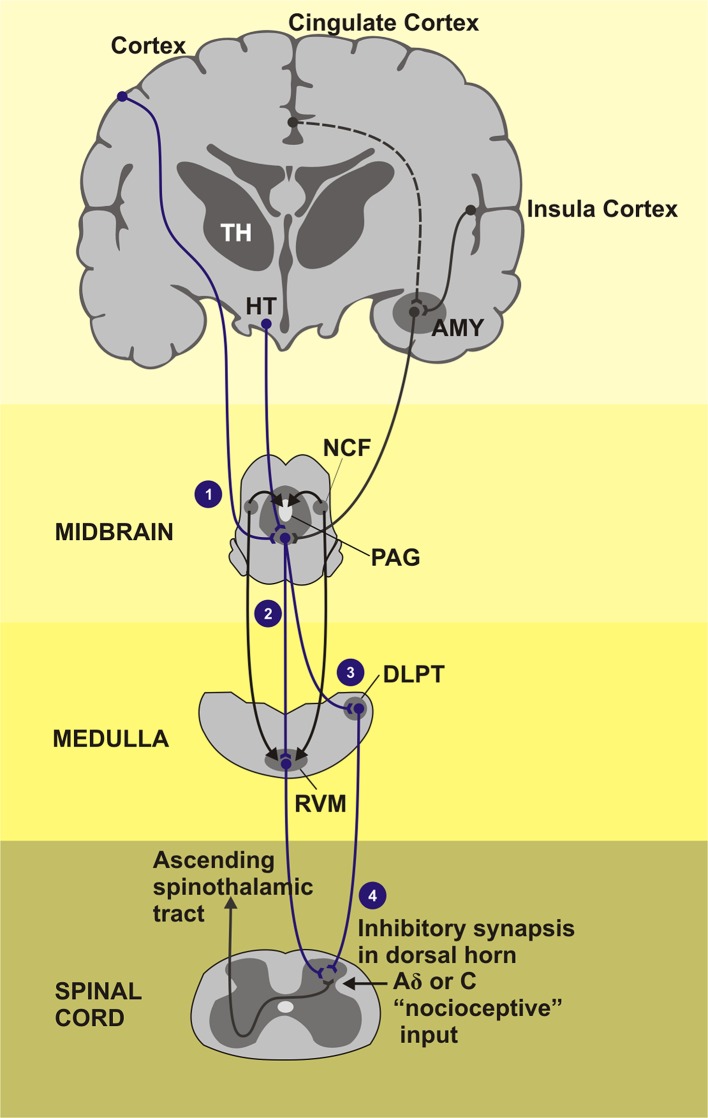
Areas of the brain that induction the descending pain inhibitory system composed by PAG-RVM-spinal cord pathway. TH, Thalamus; HT, hypothalamus; AMY, amygdala; NCF, nucleus cuneiforms; PAG, periaqueductal gray matter; DLPT, dorsolateral pontine tegmentum; RVM, rostral ventromedial medulla. (1) Cortical regions as cingulate and insula cortex as also the subcortical regions how the thalamus, hypothalamus, and amygdala project signals for the PAG, gray substance located in the midbrain, that receives stimulus and send inhibitory impulses across the medial and lateral tracts of the CNS. (2) Medial tract: inhibitory and facilitatory influence of the neurotransmitter serotonin in the pain activeness. (3) Lateral tract: dominant activity of neurotransmitter noradrenaline. (4) Fired inhibitory stimulus go down throughout dorsal horn of the spinal cord segment. The painful stimuli are sent to the second order neurons. Endorphins, noradrenaline, and serotonin, inhibitory neurotransmitters, are released activation of the inhibitory interneurons.

The top-down pathways involved in the opioid-mediated antinociception includes the modulatory effect mediated by opioid receptors found in brain structures such as the anterior cingulate cortex (ACC), midcingulate cortex (MCC), insula, PFC, basal ganglia, amygdala, hypothalamus, DLPT, PAG, RVM, and spinal cord. The role of the opioid system in inhibition of fear acquisition was blocked by the MOR antagonist naloxone in healthy subjects, as well as changes activation profile in the amygdala (Eippert et al., 2009). In the same way, pain expectations contribute to the placebo effect opioid-mediated (Benedetti, 2005; Eippert et al., 2009). For instance, the naloxone reduces the placebo effects in several cortical and subcortical areas connected to the descending pain modulatory system (e.g., rACC, PAG, RVM, and hypothalamus). The top-down pathways involved in the opioid-mediated antinociception such as PFC and ACC interact to limbic areas (e.g., amygdala and insula) that provide relief of pain aversiveness (Navratilova et al., 2015) and with the ventral striatum, that plays a central role in rewarding behavior. Remarkably, all cortical inputs converge to the PAG-RVM-spinal cord, which facilitates or inhibits nociception (Fields, 2004; Jones and Brown, 2018) (Figure 4).

### Transcranial Direct Current Stimulation: Technical Factors and Neurobiological Mechanism

The effect of non-invasive transcranial neuromodulation by tDCS can be influenced by factors such as the regions where the tDCS is applied (e.g., M1, DLPFC). This technique applies a low and continuous electric current (oftenly from 0.5 to 2 mA) transmitted directly far to the electrodes (20–35 cm^2^) on the scalp to a target area. In healthy subjects anodal tDCS, 0.5–2.0 mA resulted in similar facilitatory effects relative to sham while for cathodal tDCS, only 1.0 mA resulted in sustained excitability diminution (Jamil et al., 2017). The current runs through the scalp and reaches superficial cortical levels, acting in the membrane polarity producing neuronal modulation in these cortical regions. While anodal tDCS induces depolarization and increases excitability, and cathodal decreases the excitability in the neuronal membrane (Nitsche and Paulus, 2000; Priori, 2003; Lang et al., 2005; Nitsche et al., 2008). Anodal tDCS induces depolarization the neuronal membrane and enhances M1 excitability influencing the sensory-discriminative networks evolved in pain sensitivity processing. Anodal stimulation over DLPFC, on the other hand, has revealed beneficial effects on mood regulation, cognitive functions (e.g., decision making) and on mechanisms underlying adaptive and maladaptive emotional functioning (Dixon et al., 2017).

Studies using *in vivo* magnetic resonance spectroscopy (MRS) found that anodal tDCS reduces local cortical GABA concentration in the motor cortex (Stagg et al., 2009; Kim et al., 2014), it also increases local levels of glutamate and glutamine (Glx) in the intraparietal and prefrontal cortex measured together as combined Glx, and N-acetyl aspartate (NAA) (Clark et al., 2011; Hone-Blanchet et al., 2016). While studies that have investigated the effect of tDCS have found cathodal tDCS conduct to a significant decrease in glutamate concentration compared to s-tDCS (Witney, 2018). The tDCS effect on corticospinal excitability was blocked by the NMDA-receptor antagonist dextromethorphan (Liebetanz et al., 2002). This is compatible with activity-dependent synaptic neuroplasticity, such as long-term potentiation (LTP) and long-term depression (LTD). This experimental data give support to comprehend the link between the cumulative effect induced by repetitive sessions with the mechanisms of long-term potentiation (LTP), or with increases in the facilitation process or long-term depression (LTD). These neuroplastic mechanisms involve the effect of central neurobiological systems related to the excitability and inhibition, namely, glutamatergic and GABAergic systems, respectively. Anodal stimulation induces LTP provoked by neurotransmitters that facilitate the overture of AMPA (α-amino-3-hydroxy-5-methyl-4-isoxazole propionic acid) channels and indirectly NMDA (N-methyl-D-aspartate) receptor. The opposite occurs with a cathodic stimulation. These effects are regulated by intracellular cyclic AMP and calcium levels (Kronberg et al., 2019). Another mechanism involved in pain relief through anodic stimulation is its capacity for restoring the endogenous inhibitory system. Also, it has been showed that tDCS could reach deeper structures such as red nucleus (RN) and medial longitudinal fasciculus (MLF). tDCS can regulate cell migration (McCaig et al., 2005; Zhao, 2009), cell orientation, differentiation, and metabolism. The changes in the direction and speed of cell migration and neurite growth could be explained, at least in part, by localized shifts of intracellular Ca2+ (Palmer et al., 2000; Mycielska and Djamgoz, 2004). Thus, it facilitates the opening of voltage-dependent ionic channels and NMDAR activation (by removal of the blocking Mg2+ ions) (Pelletier and Cicchetti, 2014). The intracellular signaling pathways triggered by a substantial increase in postsynaptic calcium include activation of protein kinase C, calcium/calmodulin-dependent protein kinase II and tyrosine kinases. These molecular events result in phosphorylation of AMPA receptors in the postsynaptic membrane. Thereby, LTP is primarily expressed as an increase in AMPA receptor-mediated (Nitsche et al., 2012b). When tDCS stimulation is applied this induced LTP response, in an N-Methyl-D-Aspartate Receptor (NMDA)-dependent fashion. The LTP response is diminished in both sham and stimulated samples when NMDAR antagonists are applied (Rohan et al., 2015) and it is a fast-acting protein that allows for an influx of calcium AMPA receptors, which is ionotropic glutamate receptors that are permeable to cations, namely sodium and calcium ions (Chater and Goda, 2014). The LTP response is diminished in both sham and stimulated samples when NMDAR antagonists are applied (Rohan et al., 2015).

**Figure 5:**
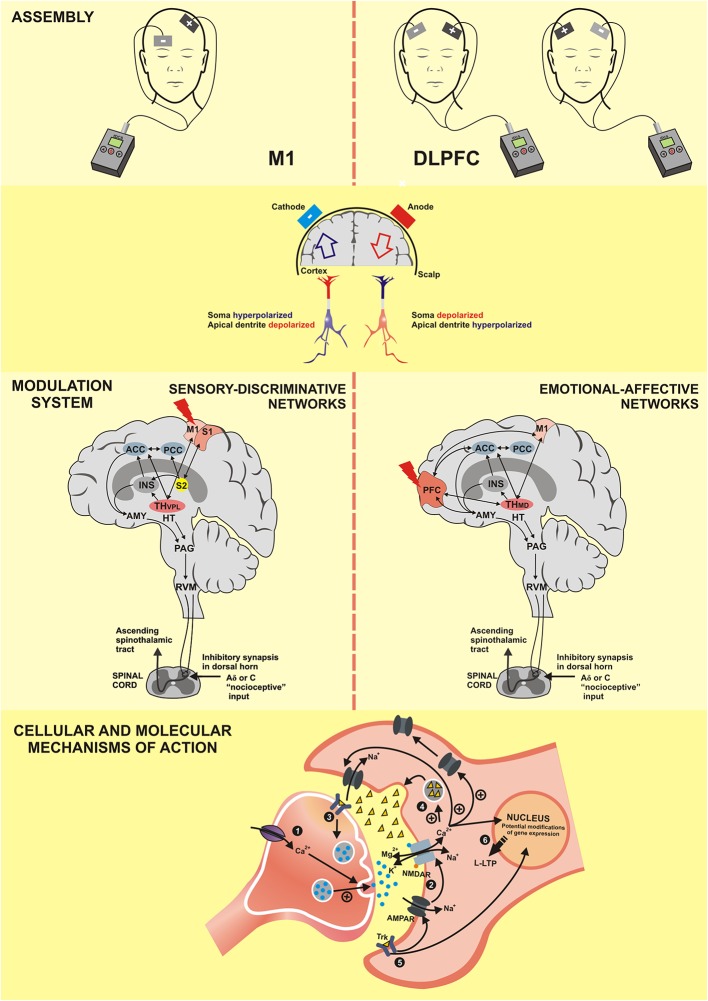
M1, Primary motor cortex; DLPFC, dorsolateral prefrontal cortex; S1, primary somatosensory cortex; S2, secondary somatosensory cortex; PFC, prefrontal cortex; ACC, anterior cingulate cortex; PCC, posterior cingulate cortex; INS, Insula; TH–VPL, thalamus-ventral posterolateral nucleus; TH-MD, thalamus-medial dorsal nucleus; HT, hypothalamus; AMY, Amygdala; PAG, periaqueductal gray matter;RVM, rostral ventromedial medulla; NMDAR, N-methyl-D-aspartate receptor; AMPAR, α-amino-3-hydroxy-5-methyl-4-isoxazolepropionic acid receptor; Trk, Tropomyosin receptor kinase; Ca^2+^, calcium; K^+^, potassium; Na^+^, sodium; Mg^2^, magnesium; +, excitatory; -, inhibitory; L-LTP, late long-term potentiation. (1) The anodal tDCS increases the intracellular Ca^2+^ flow that releases more neurotransmitters. (2) The positive regulation of neurotransmitters facilitates the openness of AMPA channels and indirectly of NMDA channels, which characterizes the long-term potentiation (LTP). (3) Activation of tropomyosin receptor (Trk) indicates the role of brain-derived neurotrophic factor (BDNF) in the anodal tDCS; its effect increases the production of the synaptic vesicles and the neurotransmitter release. (4) Increase of Ca^2+^ flow promotes the release of neurotrophic factors for the synaptic cleft. (5) Post-synaptic Trk receptor induces the late LTP (L-LTP) and favors the openness of the NMDA channels, which reinforces the L-LTP. (6) Both L-LTP and L-LTD are highly dependent on the modification of gene expression.

Another factor that contributes to this neuromodulatory action includes changes in the brain-derived neurotrophic factor (BDNF) expression. An experimental study showed that ~0.75 V/m anodal DCS increases the peak amplitude of the excitatory postsynaptic potential. However, this effect was absent in slices from BDNF knockout mice or when the TrkB receptor was blocked (Fritsch et al., 2010). In individuals expressing the BDNF Val66Met polymorphism, which affects the release of BDNF, motor skill acquisition after a 5-day tDCS treatment was significantly lower than in healthy volunteers (Pelletier and Cicchetti, 2014). In short, its effect involves several neurotransmitters, such as dopamine, acetylcholine, serotonin, GABA (Medeiros et al., 2012), and multiple mechanisms of intracellular plasticity, which affect neurotransmitters, including gene expression. The cascade of the neurobiological processes involving the tDCS effect is presented in Figure 5.

#### Transcranial Direct Current Stimulation to Treat Pain and Dysfunctional Neuroplasticity Induced by Opioids

The tDCS is a neuromodulatory technique promised to treat opioid use disorder (OUD) in comparison to pharmacotherapy and behavioral intervention strategies (Barr et al., 2011; Wing et al., 2013; Salling and Martinez, 2016). While pharmacological treatments are showing their limits, new therapeutic options that are more targeted to the dysfunctional neural circuits are being explored, such as tDCS. Although at present, the data are yet incipient to know the real effectiveness in drug-avoiding behavior (Fregni et al., 2008; Brangioni et al., 2018). It has been used to inhibit craving behavior or to control pain, hence is attractive to treating and preventing opioid dependence, respectively. Given the impact of the epidemic of opioid addiction, innovative ways are needed to help those currently addicted.

#### Effects of tDCS Applied Over M1 and DLPFC in Pain Processing

The record of stimulation for motor cortex as a scientific experiment occurred in 1780 when Galvani demonstrated the electric contraction of the frog muscle. However, the first electrical stimulation of the human brain was performed in 1874, when the motor cortex of an individual was exposed during debridement of a focus of osteomyelitis in one area of the scalp. tDCS was reintroduced as a non-invasive brain stimulating the intact human cortex in the last two decades (Nitsche and Paulus, 2000). The excitability depends on the polarity of tDCS, anodal placed over M1 and cathodal over frontal pole increase the excitability of M1 and it decreases when current flow is reversed (cathodal placed over M1; Lang et al., 2004). It is estimated that around 45% of the total current applied to the scalp produces effective modulation of regional neuronal activity in the targeted cortex (Rush and Driscoll, 1967). These changes in excitability persist beyond the time of stimulation. The tDCS effect remains stable if it is used for at least 10 min (Nitsche and Paulus, 2001; Nitsche et al., 2003). The M1 is somatotopically arranged to receive inputs from three main sources: (i) peripheral inputs via thalamic relay nuclei-somatosensory cortex, premotor cortex and sensory association areas from the cortex; (ii) basal ganglia; and (iii) cerebellum. The motor processing is overlap with those areas associated with pain neuromatrix. Anodal stimulation is associated with direct stimulation of pyramidal and cathodal is associated with an indirect stimulation of pyramidal neurons via interneurons. The processing of pain by tDCS involves different mechanism. Stimulation in M1 lead to decrease in the hyperactivity of thalamic and brainstem nuclei to result of the inhibition of these areas. Indeed, anodal tDCS of M1 induce corticothalamic inhibition of ventral posterolateral nucleus (VPL) responsible for discriminatory sensitivity and ventral posteromedial nucleus (VPM) responsible for nociceptive sensation. DLPFC stimulation decreases the activity of the midbrain-medial thalamic pathway being involved with the modulations of the structures related to the emotional perception of pain (Boggio et al., 2008). The right M1 activated by tDCS produced changes in the caudal portion of the anterior cingulate cortex, superior temporal sulcus, right parieto-occipital junction, and cerebellum. This effect indicates the functional interaction between M1 and these areas via corticocortical and cortical-subcortical connections (Lang et al., 2005).

The PFC can be divided into medial prefrontal cortex (mPFC), orbitofrontal cortex, ventrolateral, dorsolateral (DLPFC), and caudal. DLPFC neurostimulation may modulate sustained and divided attention when the tasks require workload. While the frontal and parietal areas modulate the perceptual awareness, and the right prefrontal areas may mostly control the internal focus. Anodal over the left DLPFC may reduce the perceived degree of emotional valence for negative emotional pictures (Peña-Gómez et al., 2011), and for images of anger expressions (e.g., De Raedt et al., 2010). Moreover, the left DLPFC may play a role in the upregulation of reactions to positive emotional stimuli, since anodal stimulation over this region improves the identification of positive emotional expressions (Nitsche et al., 2012a). On the other hand, the right DLPFC may be involved in the upregulation of adverse psychological outcomes. Stimulation with TMS using high frequency (i.e., excitatory) over the right DLPFC resulted in impaired attention disengagement from threat (angry faces; De Raedt et al., 2010). A similar result has been documented by Leyman et al. (2009), showing that high-frequency rTMS over the right DLPFC can reduce the ability to inhibit the processing of negative information (sad faces). Thus, stimulation of both the left and right DLPFC might counteract maladaptive plasticity of the cortical-meso-limbic network according to the purpose of treatment.

These findings indicate an important role of the PFC in pain processing. Interestingly, the activity of DLPFC has been shown to correlate negatively with the perception of pain, suggesting that the DLPFC may have a dampening effect on the activity of the midbrain-medial thalamic pathway. Thus, the DLPFC may be activated during painful states and may in turn ultimately modulate structures involved in the emotional perception of pain including the anterior cingulate cortex, insula, and amygdala (Boggio et al., 2008). A recent review expands this description, highlighting the complex connections of other regions of PFC with PAG, thalamus, amygdala and basal nuclei (Ong et al., 2019). Thus, tDCS of DLPFC may interfere with the emotional processing of pain by actively exerting control on pain perception by modulating these subcortical and cortical pathways. tDCS over prefrontal regions may exert an increased cerebral activity, as investigated in EEG technique (Maeoka et al., 2012) and can reduce pain or reduce the use of opioids in chronic pain conditions such as myofascial pain (Choi et al., 2014), multiple sclerosis (Ayache et al., 2016), and pain after lumbar spinal surgeries (Glaser et al., 2016).

tDCS bilateral right anodal/left cathodal did not change the sustained attention effect of tDCS (Heinze et al., 2014). Anodal tDCS on the right DLPFC in comparison with anodal stimulation to the left DLPFC induced higher improvement to analytical judgment and decision-making, while the logic index score diminished after left anodal stimulation. TDCS applied to the right DLPFC is known to affect executive functions (Del Missier et al., 2010) that include impulsivity control and set shifting (see Greenwood et al., 2018, for a review).

## Evidence of tDCS Impact on Pain

### Systematized Review of Evidences

In order to understand qualitative and quantitatively the evidence of the effects of tDCS on pain in chronic pain conditions, we implemented a literature review and a meta-analysis of the findings reported for pain levels after tDCS application.

### Methods

A search in PubMed, Embase, and Cochrane databases was performed in May, 2019, in Title, Abstract, and Keywords (except for PubMed, where all fields were examined). The scope of this review included clinical trials with tDCS as the main intervention for patients with chronic pain conditions. Therefore, we used the following search term (boolean operators were changed appropriately according to each database): [(“chronic pain” OR “neuropathic pain” OR “myofascial pain” OR “musculoskeletal pain” OR “migraine” OR “pain syndrome”) AND (transcranial OR NIBS OR “non-invasive brain stimulation”)].

**Figure 6:**
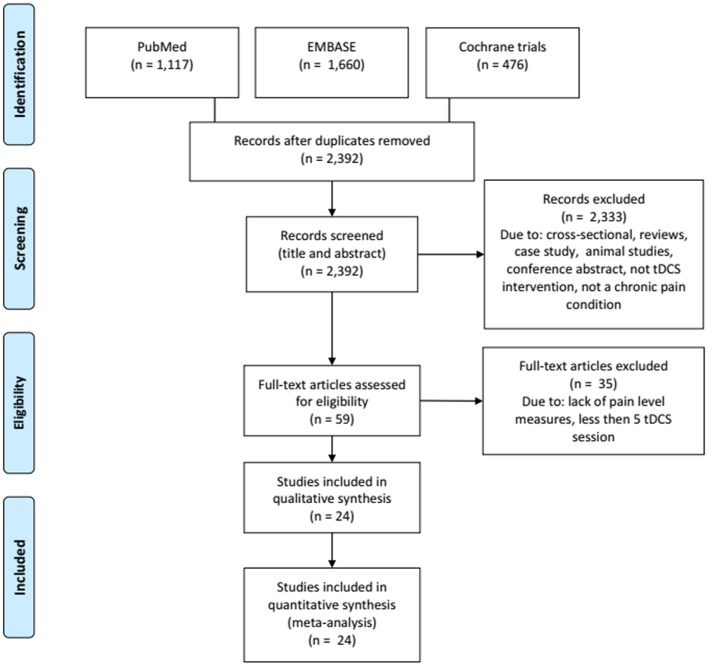
Flowchart of the systematized search.

We excluded articles if they met the following criteria: studies written in other languages than English, Portuguese, or Spanish; studies that do not reported pain as an outcome; <5 sessions of tDCS treatment; studies using other types of stimulation than direct current; reviews and case studies; cross-sectional studies; conference abstracts; tDCS protocols accepted were application in areas of the primary motor cortex (M1), dorsolateral prefrontal cortex (DLPFC), or occipital area, considering low, or high-density tDCS (HD-tDCS). In addition, studies should have Baseline and post-tDCS treatment data available (mean and standard-deviation or CI). The systematized search is presented in Figure 6.

### Results: Qualitative and Quantitative Data

Supplementary Table 1 presents methodological characteristics and main findings of the 24 studies reviewed. Efficacy was considered based on the effect sizes for the difference in Visual Analogue Scale (VAS) or Numeric Pain Scale (NPS) scores (0–10) between active and sham for the first measure after the complete treatment. Positive values favor active stimulation. Six studies had two possible comparisons with sham, and were included as two entrances in the quantitative analyses. As can be observed, publications in this field reviewed here start from 2006, since the evidence related to our interest subject stated in this period. Total sample sizes vary from 10 to 135 patients (mean = 35.1; SD = 24.9) and include several different chronic pain conditions. Considering all comparisons, number of tDCS sessions ranged from 5 to 20 (mean = 8.4; SD = 4.5) and included anodal and cathodal active stimulations, only tDCS or a combination of tDCS and other intervention (combined interventions). Device current ranged from 1 to 2 (mean = 1.7; SD = 0.5) and application time ranged from 15 to 30 min (mean = 20.2; SD = 2.3), considering in 92% of applications it lasted 20 min.

### Results: Meta-Analysis and Risk of Bias Assessment

**Figure 7:**
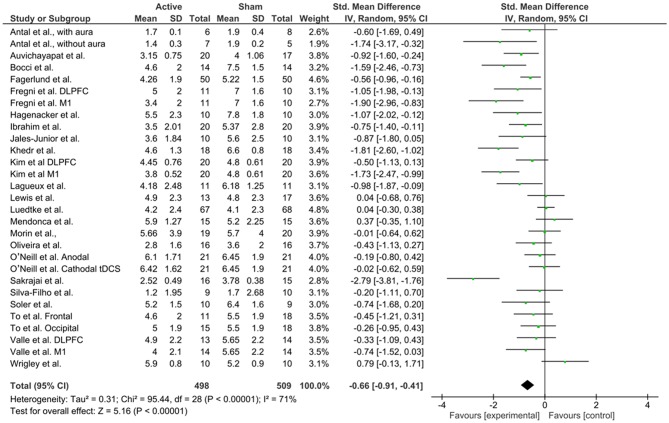
Forest plot of the effects of tDCS on pain levels for all studies reviewed (*n* = 23).

**Figure 8:**
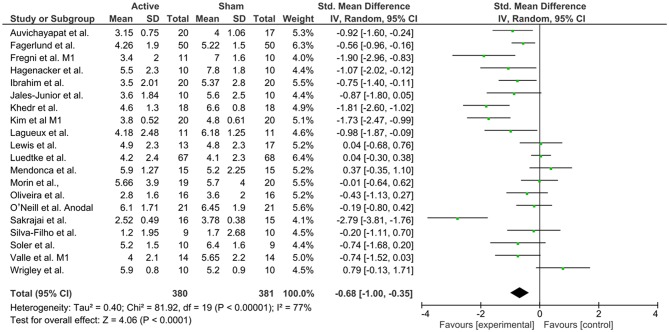
Forest plot of the effects of anodal M1 tDCS on pain levels (*n* = 20).

**Figure 9:**
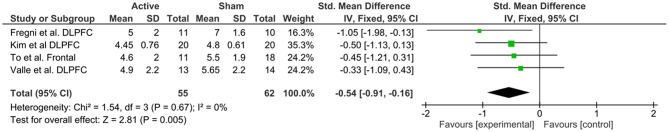
Forest plot of the effects of anodal DLPFC tDCS on pain levels (*n* = 4).

For the meta-analysis, we followed the Cochrane guidelines (Higgins et al., 2011) and used Review Manager 5 (RevMan 5.3) software to build forest plots, considering three scenarios: all studies (Figure 7), studies using anodal M1 stimulation site (Figure 8), and studies using anodal DLPFC (Figure 9), regardless of the cathode or return electrode position. Only one study was dropped from this analysis because data of treatment's end was not presented and could not be calculated, totalizing 23 works analyzed. For all studies, due to data had considerable heterogeneity (*I*^2^ = 71%), a random effects model was applied. A total of 498 patients received active stimulation and most of the studies favor active tDCS by showing a significant reduction (*P* < 0.001) of pain levels (indexed by VAS or NPS measures) when compared to sham tDCS. The standardized mean difference was −0.66 (CI 95% = −0.91, −0.41). This means a reduction of 27.26% in pain at the end of treatment for active tDCS compared to sham (95% CI; 15.89, 32.90%).

To investigate M1 efficacy, 20 studies were included. Heterogeneity was identified, therefor random effects were applied. A moderate effect size for reduction of pain levels was able to be seen (−0.68; CI 95% = −1.0, −0.35). Finally, to understand the efficacy of anodal DLPFC montages, four studies were identified. No heterogeneity was found, and then fixed effects model was applied. A moderate effect size was observed (−0.54; CI 95% −0.91, −0.16).

**Figure 10:**
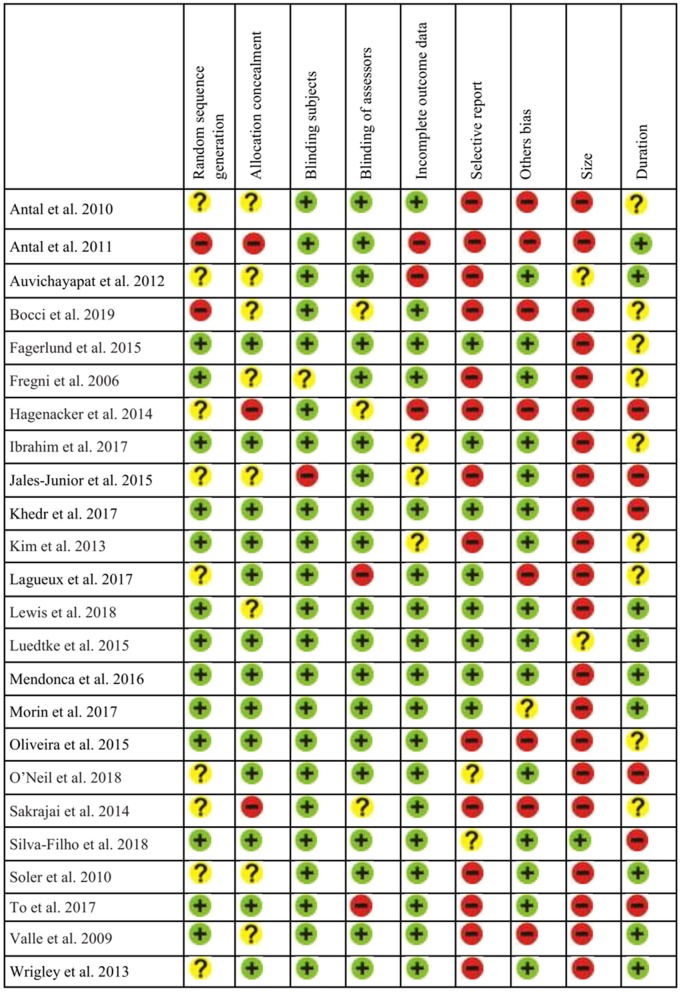
Assessment of risk of bias from the reviewed studies (*n* = 24).

For the analysis of Risk of Bias in the selected studies we examined different potential sources of bias: selection bias (random generation sequence and allocation concealment), blinding (subject and assessor), incomplete outcome data (attrition bias), selective reporting (reporting bias), other bias and possible limitations in relation to sample size and follow-up assessments. We considered the criteria from Cochrane guidelines (Higgins et al., 2011), as studies were evaluated according to (a) low risk; (b) high risk; or (c) unclear. Following the criteria of Andrew Moore et al. (2010) for evidence in studies in chronic pain, sample sizes for each group/condition were considered: (a) high risk for *n* < 50; (b) unclear (some risk) from 50 to 199; and (c) low risk for *n* > 200. Follow-up quality was judged as (a) high risk for <2 weeks after treatment; (b) unclear (some risk) from 3 to 7 weeks; and (c) low risk for 8 or more weeks. This assessment was implemented by two authors independently. In case of disagreement, a third judge decided the best option. Figure 10 presents the risk of bias assessment.

### Integrating the Findings

This systematized review was able to evidence a significant effect of tDCS in reducing the pain perception associated with chronic pain conditions compared to sham with an effect size of moderate magnitude (SMD = 0.66). Considering the diversity of clinical conditions and treatment protocols (including combined interventions), it is possible to conclude that this effect may be clinically meaningful. However, we need parsimony to generalize these findings to different contexts of chronic pain. Among several aspects related to the primary outcome measures of studies, we stress as main limitations: heterogeneity and measure the outcome based in a unidimensional measure related to severity [(e.g., pain scores on Visual Analogue Scale (VAS) and Numerical Pain Scale (NPS-0-10)]. Also, distinct pathologies with a small number of patients restrict the generalizability of the impact of treatment. Furthermore, another point to consider is that in small studies usually, the sample has more probability of being a super-selected sample. Most of the studies have as a primary outcome the pain severity by a unidimensional on measures, and they are deprived of measures of disability due to pain. In the same way, lack exploratory investigation of the influence of other comorbidities (e.g., psychiatric disorders) in the impact of tDCS. Although these studies have some limitations related to how the measures the clinical impact the tDCS effects, they are supported by data of complementary methods such as neuroimaging and neurophysiological measures. These results are essential to comprehend how the tDCS effect can change these processes; however, we need to realize that they are surrogate outcomes and were not the interest issue of this systematized review. Bellow, we discuss the studies in detail, mainly focusing on M1 and DLPFC anodal stimulations, to build up a perspective of evidence.

### Strength of Evidence for Pain Levels

According to the criteria adopted here, some studies present major methodological weaknesses, which prevents a straightforward conclusion about its findings. Therefore, tDCS for reduction of pain levels in migraine is debatable, due to problems in randomization, concealment of intervention, missing data and selective reporting (Antal et al., 2011; Auvichayapat et al., 2012). Nevertheless, they found a moderate decrease (−36.4 and −31.5%, respectively) in pain after active tDCS. For FM, seven studies were included. Although Mendonca et al. (2016) did not find a positive effect of tDCS in pain reduction when combined with an aerobic exercise (no additive effect), the other studies had a significant impact and were considered with low risk of bias (Fregni et al., 2006; Valle et al., 2010; Fagerlund et al., 2015; Khedr et al., 2017). To et al. (2017) had problems with blinding of assessors), either using M1 of DLPFC anodal tDCS stimulation site, indicating stronger evidence in this population. Concerning neuropathic pain, three studies (Soler et al., 2010; Kim Y.J. et al., 2013; Bocci et al., 2019) found reductions in levels of pain. They used different anodal stimulation sites (M1, DLPFC, and cervical for cerebellar stimulation) for various neuropathic pain syndromes. Problems in randomization (except for Bocci et al., 2019) and selective bias were found. Therefore, the evidence of an effect has its limitations.

On the other hand, Wrigley et al. (2013), Lewis et al. (2018), O'Neill et al. (2018) did not find any positive effect of tDCS for pain reduction. They did not present significant bias problems (although O'Neill et al., 2018 had a different amount of tDCS sessions for each person), which raises a conclusion of a lack of effects for the neuropathic syndromes they investigated, including spinal cord and upper limb neuropathic pain. One should consider that neuropathic pain syndromes can vary a lot in terms of etiology and neurophysiological mechanisms (Ramirez et al., 2013) and this heterogeneity, which was the case in the present review, could be associated to these findings.

Other studies evaluated tDCS effects in different pain conditions. The more substantial impact was found for myofascial pain syndrome (Sakrajai et al., 2014). The difference between reduction after sham tDCS and after active anodal tDCS over M1 for 5 consecutive days was 26.8%. However, the study had problems with concealment of allocation, and it did not present a protocol registration identified in a base of clinical trials. Also, a significant decrease on pain scores was also found for trigeminal pain (Hagenacker et al., 2014), visceral pain due to hepatocellular carcinoma (Ibrahim et al., 2018) and complex regional pain syndrome (Lagueux et al., 2018). There was no significant effect on chronic low back pain (Luedtke et al., 2015), even though this study had been considered with minimal risk of bias. It is essential to realize low back pain is a complaint of several distinct pathological insults, including trauma, infection, inflammation, and systemic disease such as cancer, etc. This condition can be classified broadly into four pain states: nociceptive, inflammatory, neuropathic, and centralized/dysfunctional. Therefore, this heterogeneity might explain the lack of results. Another study (Antal et al., 2010) also found significant reductions for pain severity (35% of change from baseline for the active tDCS group), although it has included 12 patients with various pain syndromes, encompassing fibromyalgia, chronic back pain, trigeminal neuralgia, face pain, arthrosis, post-stroke pain, and polyneuropathy.

Bringing into account this scenario, it could be suggested that anodal tDCS may be useful in pain reduction. However, further studies should consider the mechanisms underlying clinical conditions and the pain state to personalize the therapeutic approaches. Concerning the type of protocol, recent proposals discuss a spatial-mechanistic framework (Yavari et al., 2017) that should be considered when mounting tDCS, reasserting the importance of electrode size and positioning. Besides, as the guidelines for quality of evidence has evolved, researchers should pay attention to some limitations found here. To the risk of bias, the major problem was related to small sample sizes for each treatment arm. Only two studies in our review reached a moderate sample size as recommended by the IMPACT guideline for chronic pain studies; the others had small sample sizes. Follow-up assessment and research protocol registration were also of some concern and should be considered in future investigations.

### Effect According to Site of Stimulation (M1 or DLPFC)

There is a contemporary debate about the best site for anodal stimulation for pain syndromes. The M1 region has been classically used and, as described on section Introduction, it is an important part of the pain neuromatrix, with efferent connections to many subcortical regions. The 20 trials that used this montage observed a SDM equal to −0.68 (CI 95% = −1.00, −0.35), which represents a moderate effect of active compared to sham tDCS. On the other hand, we analyzed separately anodal DLPFC effects, which was investigated through four studies, three of them in fibromyalgia patients, and yielded an effect of −0.54 for active compared to sham in reducing pain levels (CI 95% = −0.91, −0.16). Fregni et al. (2006) was the only study that not found a significant effect of DLPFC on pain, although the impact on depression and cognitive performance were reported.

This scenario indicates, at the first place, that DLPFC has a potential effect in pain levels, although through different neural mechanisms, even though a small number of studies has been implemented so far. Second, the effects of the DLPFC may be more pronounced for other clinical and behavioral measures, as To et al. (2017) for fatigue effects and Fregni et al. (2006) for emotional-affective and cognitive measures.

### Evidence for Other Clinical Outcomes

Although the discussion so far has focused on the level of pain (or severity of pain), it is crucial to be aware that this outcome counts only partially to the health condition and quality of life for patients with chronic pain. According to IMMPACT, it is essential to consider besides pain level, the physical disability, emotional functioning, participants' ratings of satisfaction, symptoms and adverse events, and participants' disposition (Edwards et al., 2016). Thereby, we also considered in this review other clinical measures that were investigated. As described in Supplementary Table 1, many studies also investigated depression and anxiety symptoms, and other psychiatric symptoms and functional capacity or how much pain impact daily life activities. Some studies also included psychophysical measures of pain, such as pain threshold and tolerance. A few have examined patient and physician global impression of the treatment. IMMPACT suggested outcomes are not observed in many studies, probably due to specific objectives of some of them, which includes neurological, physiological and biological measures, as well as criteria specific to the condition investigated, which would result in excessive parameters to be analyzed. Nevertheless, this points out to effects of tDCS in other dimensions of pain, which requires further review and meta-analysis.

### Considerations of tDCS Application for OUDs and Opioid Use for Chronic Pain Syndromes

Few studies investigated the application of tDCS to counter-regulate the dysfunction in the DPIS induced by opioids. In healthy subjects in an experimental model, we showed that the tDCS applied over M1 blocked the dysfunction caused by remifentanil on the inhibitory pathways or up-regulation of the pain-facilitating pathways (Braulio et al., 2018). This effect of tDCS was demonstrated to prevent both the disengagement of the DPIS and the summation effect on pain scores during the cold pressor test (Braulio et al., 2018). When focusing on opioid dependence, a recent review (Gallucci et al., 2019) found only one study that used tDCS. Wang et al. (2016) reported a reduction of 36.7% in craving for heroin after watching a real video of heroin use in individuals who were addicted in the past and were in abstinence for at least 1.5 years. Perhaps more interestingly was Gallucci et al. (2019) findings related to pain and analgesic use in postoperative contexts, based on seven studies. Although only one study in postoperative acute pain found reductions in pain perception after the procedure hallux valgus surgery (Ribeiro et al., 2017), there were significant reductions in patient-controlled analgesia (PCA) method in six studies (Borckardt et al., 2011, 2013, 2017; Dubois et al., 2013; Glaser et al., 2016; Khedr et al., 2017). This effect ranged from small (0.3) to large (0.95). Even though these results have been indicated an important reduction in opioid use for certain conditions, to the best of our knowledge, it is unknown the effect of tDCS to reduce the opioid in chronic pain treatment use and to improve chronic refractory pain in patients with opioid use in high doses and a long-term.

Use of opioids are mandatorily associated with the severity of pain reported, considering it is the leading information in medical routines for the clinician to administer opioid drugs. Psychological factors are also linked to OUD and addictive behaviors (Darnall, 2012). It has been reported a bidirectional relation between depression and opioid in chronic pain women. Anxiety disorders are associated with more substantial use of opioids. On the other hand, people with higher pain catastrophizing, a psychological construct related to magnified, ruminative, and helplessness thoughts have been linked to inadequate response to opioid treatment. Thus, these measures need to be considered in studies which focus on the reduction of opioid use. Not only in OUD cases these measures might be relevant, but also for opioid analgesics use in non-cancer chronic pain conditions. Richards et al. (2018) found patients with lower back pain using opioids for more than 3 months had a worse attention performance and few pain self-efficacy beliefs than patients not taking opioids. Problematic use of opioids in chronic pain condition can affect up to 81% of patients, and addiction can range from 8 to 12% (Vowles et al., 2015), which signals to a significant health problem. Besides, the FDA recommends that the better evidence on the severe risks of misuse and with long-term use of opioids, predictors of opioid addiction such as a history of previous dependence, psychiatric diagnosis, benzodiazepine dependence, etc. Although the use of tDCS to reduce cravings scores in OUDs is yet an emergent research question, its effect finds plausibility to affect brain regions, which are triggers for activation of dopaminergic circuitry (Strafella et al., 2001; Pogarell et al., 2006).

In the same way, it may change the synaptic plasticity in the reward system (Pascoli et al., 2014). In addition, the activation of the frontal cortical region leads to changes in cortical areas connected with behavioral inhibition or decision making (Fecteau et al., 2014; Ouellet et al., 2015) and may stimulate the subcortical regions related to the motivation (Botvinick and Braver, 2015). However, further brain imaging studies are required to elucidate the underlying mechanisms following tDCS treatment to addiction. Aligned with data, studies found that acute anodal tDCS over left DLPFC increased the ability to resist smoking (Falcone et al., 2016). The left cathodal and right anodal tDCS over bilateral DLPFC in a single tDCS session reduced the activation of cingulate cortex in crack-cocaine users during a visual test to assess the cortical activation when a drug-user was exposed to a condition that could get asses to drug (Conti and Nakamura-Palacios, 2014), and tobacco intake (Fecteau et al., 2014). Furthermore, the tDCS over the bilateral frontal-parietal-temporal (FPT) area has been found to reduce cue-induced craving in nicotine dependents (Meng et al., 2014).

## Perspectives for Future Studies Considering the Impact of tDCS on DPMS as an Approach to Treat the OHI

### Future Direction

The scientific advance of neuroscience has witnessed a huge leap forward the comprehension of the mechanistic underpinnings of pain. Also, the number of treatment targets has grown substantially. However, despite these advances, at present, in the clinical practice, we have not yet been able to apply an evidence-based approach to chronic pain treatment that reflects mechanistic understanding, and the clinical management remains an empirical and often unsatisfactory journey for patients. In this review, we propose an approach of usage of transcranial neuromodulatory tools, specifically tDCS, for the management of pain symptoms in chronic refractory pain associated with OUD. We discussed the molecular mechanisms and methods of assessment and management still fall well short, as well as the opioid effects on pain pathways and how they theoretically can disrupt the function of descending pain inhibitory system. Our review struggles to show that common difficulties when it comes to the treatment of pain condition in OUD in clinical practice is related to the incipient understanding pathophysiological pain mechanisms and how we could drive a rational treatment choice. The current pain classification is based mainly on descriptors, signs, and areas of the body where pain symptoms are topically referred to, combined with information regarding anatomical pathology (e.g., MRI evidence of spinal stenosis). Rarely it is done in a perspective that integrates a biopsychosocial framework. Nevertheless, substantial improvements in chronic pain management could be possible if a more strategic and coordinated approach could identify specific mechanism, aligned with a biopsychosocial perspective in accordance with the ACTTION-American Pain Society Pain Taxonomy (AAPT), which include the following dimensions: (1) core diagnostic criteria (e.g., symptoms, signs, diagnosis tests, chronic pain condition, etc.); (2) standard features (e.g., location, temporal qualities, descriptors, fatigue, numbness, etc.); (3) medical comorbidities (e.g., major depression); (4) neurobiological, psychosocial, and functional consequences; and (5) putative neurobiological and psychological mechanisms, risk factors, and protective factors (central sensitization, dysfunction of DPIS, somatosensory amplification) (Fillingim et al., 2014). We integrate the consequence of chronic pain treatments based on opioids, following recommendations of guideline for opioid therapy in chronic non-cancer pain, which recommended optimization of non-opioid pharmacotherapy (anticonvulsants, antidepressants) and non-pharmacologic therapy (electroacupuncture, physical activity, TMS, tDCS, cognitive behavioral therapy, mindfulness, etc.), rather than a trial of opioids (Busse et al., 2017). Their modest effect on pain reduction supports the rationale to consider alternative theories to opioids in non-cancer pain, and on functional improvement in comparisons with no add-opioid to therapy (Busse et al., 2017). Among several reasons that justify the short-term efficacy of opioids on long-term treatment, it is that the criteria used to define the therapy have generally failed to account the pathophysiology of pain, which focus in peripheral markers of anatomic pathology and/or disease severity. In perspective, this integrative view aims to consider the therapeutic effects by impacting specific mechanisms, such as neurobiological and/or psychosocial. That is, the diagnostic criteria for specific chronic pain disorders should be determined based on mechanistic and diagnostic evidence, rather than historical precedent or theoretically expectancies. Aligned with this perspective, even in the use of transcranial neuromodulatory approaches such as tDCS, we could consider dimension to the neurobiological and psychosocial factors that contribute to raise and to maintain the chronic pain conditions. Considering the extensive review here, it is plausible to conclude that the target (or a prioritized target) of treatment needs to be defined before tDCS sessions are applied. For instance if therapy focus on the impact of pain severity on disability due to pain the major evidence points out to apply anodal tDCS over M1. If the major concern are emotional aspects related to pain, anodal tDCS over the left DLPFC would be the indicated montage. If the purpose is to reduce craving and addictive behaviors, the diencephalic montage where anodal tDCS is applied to right DLPFC may be the first option. Specifically, a stimulus in DLPFC can affect response inhibition, and this area is involved in craving behavior, which includes substance use disorder and behavioral addiction, modulating cortical excitability. The methodology more often used for this is right prefrontal anode and left the prefrontal cathode and from 1 to 2 mA for 20 min (Sauvaget et al., 2015). Also, the M1 is likely more effective to improve the dysfunction of descending pain modulatory systems. In short, tDCS could effectively improve the disinhibited state of cortical neural circuits, resulting in better control of both pain and pain-related emotions. Importantly, sex, age, premorbid psychosocial functioning, duration of pain, its local or widespread pain, medicines, etc. are factors that should be considered in the therapeutic plan. Accordingly, we propose that further studies follow a framework for pain management, which would define the pain state, pain mechanism, and molecular target. Such an approach could help as the foundation for a new era of precision for pain therapy. They should identify the pain state, considering that more than one can be present (e.g., neuropathic, nociceptive, inflammatory, and dysfunctional), to in the diagnosis the general pain mechanism (nociceptive transmission, peripheral sensitization, ectopic activity, central sensitization, and central disinhibition) and molecular targets (e.g., BDNF Val66Met, MAO, and COMT Val158Met genotypes, etc.).

According to a tool that we developed, in the validation study, the method is safe with effects like observed in studies that the stimulation was administered in medical centers. According to a tDCS device developed in our research group, the home-use method was found to be safe with effects like observed in studies that the stimulation was administered in medical centers. This device of tDCS that patients can apply themselves allows programming the parameters of the stimulus according to predefined by the clinician with a lock system to avoid changes by other individuals. Besides, it will enable monitoring the adherence to treatment by recording the time of use, impedance, and current flow. The device can offer the possibility to be to self-administration at home. Additionally, it provides an option to be prepared with the type of stimulation by a researcher not involved in the patients' assessment to allow an improvement in the quality of blinding. In short, we present evidence for use in future research, including psychosocial-social characteristics, sleep patterns, response to test to pain (pain threshold, summation test, etc.), endogenous pain-modulatory processes, and a possible response for the pharmacologic challenge.

### Limitations

The main limitation in this review is the number of articles and the critical heterogeneity among them precluding drawing more firm conclusions regarding the use of tDCS to prevent and treat opioid dependence in chronic pain. However, this is an important emerging research area that can have a significant impact on public health, given the opioid epidemic and given the lack of cost-effective alternatives. However, several aspects emerge, mainly by the risk of bias analysis, small sample size, heterogeneity, which reduces the strength of the recommendation grade. The small patient series indeed hamper the quality of evidence investigated, in the same way, are the lack of adequate and accurate blinding. It is necessary more data to have a more clear idea about the ability to maintain the therapeutic effects over time since there is limited data related to long-lasting stimulation protocols as well as lack of data regarding the influence of age and time of day that the stimulation was applied.

Although the research with tDCS for the treatment of OUD shows useful perspectives, these results are preliminary and further research is needed. Further studies are required to understand differences in efficacy according to the targeted brain region. Furthermore, another gap that persists to be investigated is the polysubstance use disorders and co-occurring psychiatric disorders. Finally, we need studies to understand the potential benefits of concurrent therapies (pharmacological, behavioral) or the use of brain stimulation to prevent a participant's chances of becoming abstinent. Also, another gaps in the literature are worth noting, including the lack of optimal brain stimulation parameters in OUDs and data related to the effect the tDCS combination studies with approved pharmacological, and behavioral treatments.

## Conclusion

In comparison with sham stimulation, tDCS applied over M1 demonstrated a superior effect in reducing pain severity in non-cancer chronic pain conditions. Although the impact of tDCS over the left DLPFC indicates beneficial effects, the number of studies is smaller and more investigations are needed to allow definitive conclusions. However, in most of the studies, the number of patients is <50, and follow-up evaluations are performed <3 months. In overall, these results give perspectives that top-down neuromodulatory effects of tDCS are a promising approach to improve management in refractory non-cancer chronic pain and to enhance dysfunctional neuronal circuitries involved in the DPIS and other pain dimensions, such as emotional and cognitive. However, further studies are needed to determine individualized protocols according to a biopsychosocial perspective.

## Author Contributions

WC, LR, and GB elaborated section Introduction. RA and WC elaborated all figures and schemes presented in section Introduction. MZ, LR, CA, and RA reviewed the studies included in the systematized review of tDCS efficacy on pain. MZ and LR synthesized and described studies characteristics and main findings of the review. MZ and WC elaborated section Evidence of tDCS Impact on Pain, including the meta-analysis. WC, IT, and FF elaborated section Perspectives for Future Studies Considering the Impact of tDCS on DPMS as an Approach to Treat the OHI. CA and MZ reviewed and included references. WC conceived study's proposal and made the final review.

### Conflict of Interest

The authors declare that the research was conducted in the absence of any commercial or financial relationships that could be construed as a potential conflict of interest.

## Abbreviations

5-HT1A: serotonin 1A receptor
AAPT: ACTTION-American Pain Society Pain Taxonomy
ACC: cingulate cortex
AMPA: α-amino-3-hydroxy-5-methyl-4-isoxazole propionic acid
ATP: adenosine triphosphate
BDNF: brain derived neurotrophic factor
CCL2: monocyte chemotactic protein
CNS: central nervous system
COMT: catechol-O-methyltransferase
CSP: cortical silent period reduction
CSS: central sensitization syndrome
CXCL1: chemokine CXC 1
CXCL-12: chemokine (C-X-C motif) ligand 12
CxCRs: chemokine receptors
DLPFC: dorsolateral prefrontal cortex
DLPT: dorsolateral pontine tegmentum
DMN: default mode network
DOR: δ-opioid receptors
DPIS: descending pain inhibitory system
DSM-IV: Diagnostic and Statistical Manual of Mental Disorders
FDA: Food and Drug Administration
FPT: frontal-parietal-temporal
GABA: γ-Aminobutyric acid
Gi: Gi protein, alpha subunit
Glx: glutamate-glutamine
GPCR: protein-coupled receptor
GRK: G protein receptor kinase
HD-tDCS: high-definition transcranial direct current stimulation
HIO: hyperalgesia induced by opioids (HIO)
IASP: International Association for the Study of Pain
ICI: short intracortical inhibition decrease
iG-protein: immunoglobulin
IL-10: interleukin 10
IL-1β: interleukin 1β
IL-6: interleukin 6
IMMPACT: Initiative on Methods, Measurement and Pain Assessment in Clinical Trials
KCC2: neuron specific potassium-chloride co-transporter
KOR: κ-opioid receptors
LTD: long-term depression
LTP: long-term potentiation
MAO: monoamine oxidase
MCC: midcingulate cortex
MLF: medial longitudinal fasciculus
MOR: μ-opioid receptors
mPFC: medial prefrontal cortex
MRI: magnetic resonance imaging
MRS: magnetic resonance spectroscopy
MT: motor evoked potential
NAA: N-acetyl aspartate
NIBS: non-invasive brain stimulation
NMDAr: N-methyl-D-aspartate receptor
NPS: numeric pain scale
NSAIDs: nonsteroidal anti-inflammatory drugs
OIH: opioid-induced hyperalgesia
ORL-1: opioid receptor-like 1
OUD: opioid use disorders
P2X4: purinergic ionotropic receptors 4
P2X7: purinergic ionotropic receptors 7
PAG: periaqueductal gray matter
PCA: patient-controlled analgesia
PFC: prefrontal cortex
PKC: protein kinase C
RN: red nucleus
rTMS: repetitive transcranial magnetic stimulation
RVM: rostral ventromedial medulla
SNRIs: selective norepinephrine reuptake inhibitors
SSRIs: serotonin reuptake inhibitors
tDCS: transcranial direct current stimulation
TLR4: Toll 4 type receptors
TMS: transcranial magnetic stimulation
TNFα: tumor necrosis factor alpha
TrkB: tropomyosin receptor kinase B
VAS: visual analog scale
VPL: ventral posterolateral nucleus
VPM: ventral posteromedial nucleus.
