# Supplementary material for: Transcranial Direct Current Stimulation to Improve the Dysfunction of Descending Pain Modulatory System Related to Opioids in Chronic Non-cancer Pain: An Integrative Review of Neurobiology and Meta-Analysis
Source: Front Neurosci. 2019 Nov 18;13:1218. doi: 10.3389/fnins.2019.01218 (PMC6876542; doi:10.3389/fnins.2019.01218)
Supplement: Supplementary file 1 [file Table_1.pdf]

Table 1. Methodological characteristics and main findings of the reviewed studies (n=24)

Authors (year)	Study design	Sample size (n)	tDCS intervention				Assessent points	Instruments of assessment	Main findings	Percentage of change in VAS pain from baseline
**Medical condition**			**Current intensity / Electrode size / Current density (mA/cm²)**	**Session duration (mins)**	**tDCS montage**	**Number of sessions and treatment protocol**				
**Migraine**										
Antal et al., 2011	Parallel RTC	Total: **26**<br><br>With aura<br>Cathodal: 6<br>Sham: 8<br><br>Without aura<br>Cathodal: 7<br>Sham: 5	1 mA<br><br>5x7cm<br><br>*J* = 0.029	15’	Cathode over Oz<br><br>Anode over Cz	Total: **18**<br><br>3 times/wk during 6 wks (sham for 3 wks/cathodal for 3 wks)	During 8 wks before treat.<br>During 8 wks after treat.	Intensity of migraine attacks (0 – 3)<br>Other information related to migraine symptoms<br>Adverse effects	Decrease in migraine related days, intensity of pain during the attacks, and duration of the attacks in the active group, but not between groups; decrease pain intensity in a 0–3 pain score. Participants with aura presented better outcomes.	With aura:<br>Active: -5.0%<br>Sham: +11.8%<br><br>Without aura:<br>Active: -36.4%<br>Sham: -5.0%
Auvichayapat et al., 2012	RCT parallel	Total: **37**<br><br>Active: 20<br>Sham: 17	1 mA<br><br>35cm²<br><br>*J* = 0.029	20’	Anode over left M1<br><br>Cathode over SO contral.	Total: **20**<br><br>Daily for 20 days	4 wks before treat.<br>12 wks after treat.	VAS pain<br>Frequency of attacks<br>Abortive and analgesic medication	Decrease pain intensity in the active relative to sham group at the 4 and 8 weeks follow-up points, while there was no statistically significant reduction at 12 weeks	Active: -31.5%<br>Sham: -4.1%
**Fibromyalgia**										

Fagerlund et al., 2015	RCT parallel	Total: **50**<br><br>Active: 25<br>Sham: 25	2mA<br><br>5x7 cm<br><br>*J* = 0.057 mA/cm2	20'	Anodal left M1<br><br>Cathode over SO contral.	Total: **5**<br><br>Daily for 5 days	Pretest<br>After day 1, 2, 3, 4 and 5<br>Posttest (after 30 days	NRS (pain)<br>FIQ<br>HADS<br>SCL-90R<br>SF-36	Decrease in pain intensity after the 4th day of intervention, but small clinical significance. Small increase in FIQ and SCL- 90 in the active group	Active: -13.6%<br>Sham: -1.7%
Jales Junior et al., 2015	Sham-controled RCT	Total: **20**<br><br>Actvive:10<br>Sham: 10	1mA<br><br>3x5cm<br><br>*J* = 0.067	20'	Anode over left M1<br><br>Cathode over SO contral.	Total: **10**<br><br>Once/wk for 10 wks	Before treatm.<br>After treatm.	VAS pain<br>SF-36<br>FIQ<br>Tender points evaluation with algometer	M1 tDCS was effective for therapeutic pain control and improved quality of life. Significant changes in imaging with decreased biparietal hypoperfusion after stimulation.	Active: -40.5%<br>Sham: -16.4%
Khedr et al., 2017	RCT	Total: **36**<br><br>Actvive: 18<br>Sham: 18	2mA<br><br>24cm²<br><br>*J* = 0.083	20'	Actv. anodal left M1<br><br>Cathode over sholder<br><br>sham	Total: **10**<br><br>Daily for 10 days	Before treatm.<br>After 5th sessions<br>After 10th session<br>After 1 month<br>After 2 months	VAS pain<br>Opioid consumption and other analgesics<br>LANSS	M1 tDCS was effective on all outcome measures for pain and mood; BEL increased after treatment (both anodal and sham groups) showing a negative correlation with all other outcomes in the anodal tDCS group	Active: -38.2%<br>Sham: -17.5%
Mendonca et al., 2016	sham-controlled RCT in add-on to aerobic exercise	Total: **45**<br><br>1) tDCS/AE (aerobic exerc.): 15<br>2) AE only: 15	2mA<br><br>35m²<br><br>*J* = 0.057	20'	Anode over left M1<br><br>Cathode over SO contral.	Tota: **5**<br><br>Daily for 5 days<br><br>AE 3 times a wk for 4 wks	Before treatm.<br>After day 1, 2, 3 and 4	Visual Numeric Scale (VNS) Pain<br>VNS anxiety<br>Algometer - Pressure Pain Threshold<br>SF-36<br>BDI	The combination intervention had a significant effect on pain, anxiety and mood	Active: -18.0%<br>Sham: -23.5%

		3) tDCS only: 15						Transcranial Magnetic Stimulation – cortical excitability<br>Adverse Effects		
To et al., 2017	Prospective, single-blinded, placebo controlled, randomized, parallel-group study	Total: **42**<br><br>Occipital anode: 15<br>Bifrontal: 11<br>Sham: 18	1.5mA<br><br>35cm²<br><br>*J* = 0.043	20'	- Occipital: left and right C2 nerves dermatomes (i.e. left anode, right cathode)<br>- Bilateral: anode left DLPFC cathode right DLPFC	Total: **8**<br><br>2 sessions per wk, during 4 wks	Before treatm.<br>Immediately after treatm.	NRS pain<br>PCS<br>Modified Fatigue Impact Scale	Repeated sessions of C2 tDCS significantly improved pain, but not fatigue, whereas repeated sessions of DLPFC tDCS significantly improved pain as well as fatigue	Occipital: -29.3%<br>Frontal: -32.4%<br>Sham: -8.3%
Fregni et al., 2006	Randomized, sham-controlled, parallel groups, proof of principle study	Total: **31**<br><br>M1: 11<br>DLPFC: 11<br>Sham: 10	2mA<br><br>35cm²<br><br>*J* = 0.057	20'	M1:<br>Anode left M1<br><br>DLPFC:<br>anode left DLPFC<br><br>Cathode SO contralat.	Total: **5**<br><br>5 consecutive days	Before treatment (2 wks observation)<br>After each day of treatment<br>After 21 days	VAS pain<br>Clinician global impression (CGI) and patient global assessment (PGA)<br>Number of tender points<br>FIQ<br>SF-36<br>BDI<br>VAS anxiety<br>MMSE<br>Stroop test<br>digit span forward and backward<br>Simple reaction time task	Anodal tDCS of the M1 induced significantly greater pain improvement compared with sham stimulation and stimulation of the DLPFC, still significant after 3 weeks of followup, and small positive impact on quality of life	Active (M1):<br>-60.0%<br>Active (DLPFC):<br>-37.5%<br>Sham: -5.4%

Valle et al., 2010	RCT parallel	Total: **41**<br><br>Active M1: 14<br>Active DLPFC: 13<br>Sham: 14	2 mA<br><br>5 x 7 cm<br><br>*J* = 0.057	20'	M1: anode left M1<br><br>DLPFC: anode left DLPFC<br><br>Cathode SO contralat.	Total: **10**<br><br>For 10 consecutive days	Baseline assessment<br>After end of stimulation<br>After 30 days<br>After 60 days	VAS pain<br>FIQ<br>BDI<br>STAI<br>GDS<br>MMSE<br>Adverse events	Both M1 and DLPFC displayed improvements in pain and quality of life, but only M1 resulted in long-lasting clinical benefits as assessed at 30 and 60 days after the end of treatment.	Active (M1):<br>-33.3%<br>Active (DLPFC):<br>-30.0%<br>Sham: -8.9%
**Neuropathic pain**										
O'Neill et al., 2018	RTC crossover	Total: **21**	1.4 mA<br><br>5×5 cm<br><br>*J* = 0.056	20'	1) Anode over M1 contral to pain (cathode over SO)<br><br>2) Cathode over M1 contral to pain (anode over SO)	**Varied**<br><br>From 0 to 7 sessions each protocol (anodal, cathodal, sham)	Last assessment: day 14th	Usability of patient-administered tDcs<br>Patient compliance with device<br>NRS pain<br>SF-36<br>HADS<br>MMSE	Did not show a beneficial effect of tDCS in pain, anxiety, depression or quality of life	Active: -5.0%<br>Cathodal: +3.8%<br>Sham: -0.4%
Lewis et al., 2018<br><br>Upper limb neuropathic pain.	Double-blinded, randomized controlled trial.	Total: **30**<br><br>Active: 13<br>Sham: 17	1 mA<br><br>7x5 cm<br><br>*J* = 0.029	20'	Anode over left or right M1 (contral. To affect limb)<br><br>Cathode over SO contralateral	Total: **5**<br><br>Daily for 5 days	Baseline<br>Aftter 7 days<br>After 21 days<br>After 56 days	BPI<br>SF-MPQ2<br>Disorders of the Arm, Shoulder, and Hand questionnaire (DASH)<br>QST	The outcome measures were re -assessed 1, 3, and 8 weeks following the intervention. rating. Secondary outcome measures included the BPI interference score, Short-Form McGill Pain Questionnaire 2 (SF -MPQ2), Disorders of the Arm, Shoulder, and Hand questionnaire (DASH), and QST	Active: -21.0%<br>Sham: -4.0%

									of the nociceptive system.	
Soler et al., 2010<br><br>Spinal cord injury	RCT parallel	Total: **40**<br><br>1) active tDCS + visual illusion (VI): 10<br>2) a-tDCS: 10<br>3) sham-tDCS + VI: 9<br>4) placebo: 10	2 mA<br><br>35cm²<br><br>*J* = 0.057	20’	Anode over M1 left or right<br><br>Cathode over SO contral.	Total: **10**<br><br>Daily for 10 days	Before treatm.<br>Immediately after<br>After 2 wks<br>After 4 wks<br>After 12 wks	NRS pain<br>Neuropathic pain symptom inventory<br>BPI<br>NRS anxiety<br>Patient global impression of change<br>Adverse effects	Decrease in general pain intensity and in subtypes of neuropathic pain: dysaesthesias. tDCS+VI	Active (a-tDCS + VI): -30.7%<br>Sham (s-tDCS + VI): -11,1%
Wrigley et al., 2013<br><br>Spinal cord injury	RCT crossover	Total: **10**	2 mA<br><br>5x7cm<br><br>*J* = 0.057	20’	Anode over M1 left or right<br><br>Cathode over SO contral.	Total: **5**<br><br>Daily for 5 days	Initial<br>Pre-treat.<br>During treat.<br>4 wks after treat.<br>Pre-treat. (after cross-over)<br>4 wks after cross-over<br>After 4 months	American Spinal Injury Association Impairment Scale<br>Neuropathic Pain Scale<br>BDI<br>Side effects	Not alter significantly after each of the 5 active treatment sessions as well as after 4 weeks<br>of follow-up and 6 months of follow-up.	Active: +5.3%<br>Sham: +2.0%
Bocci et al., 2019<br><br>Phantom limb pain	crossover, double-blind, sham-controlled design	Total: **14**	2 mA<br><br>35cm²<br><br>*J* = 0.08	20’	Anode median line 2 cm bellow inion<br>Cathode over right shoulder	Total: **5**<br><br>Daily for 5 days	Baseline<br>End of stimulation wk<br>After 2 wks<br>After 4 wks	VAS phantom pain, movements and sensations<br>Laser-Evoked Potentials (LEPs)	Anodal polarization significantly dampened LEP amplitudes, reduced paroxysmal pain, non-painful phantom limb sensations and phantom limb movements	Active: -41.0%<br>Sham: -5.0%

Kim et al., 2013 (b)<br><br>Diabetic Polyneuropath y	Randomized, Sham Controlled Parallel Trial	Total: **60**<br><br>3 groups:<br>1) tDCS M1: 20<br>2) tDCS DLPFC: 20<br>3) tDCS sham: 20	2mA<br><br>25cm²<br><br>*J* = 0.08	20’	G1: Anode left M1 (C3)<br>G2: Anode left DLPFC (F3)<br>Cathode SO contralateral both	Total: **5**<br><br>Daily for 5 days	Before treatm.<br>After day 1, 2, 3, 4 and 5<br>After 2 wks<br>After 4 wks	VAS pain<br>Clinical Global Impression (CGI)<br>BDI<br>Pain threshold (PT) using algometer<br>Glycated hemoglobin (HbA1c) concentration<br>Adverse effects	tDCS over the M1 produced immediate pain relief, and relief 2- and 4-week in duration, as well as increase in pain threshold, global impression and decrease in anxiety, sleep problems and depression.<br>DLPFC stimulation decreased VAS and increased patient global impression.	Active (M1):<br>-34.0%<br>Active (DLPFC):<br>-22.0%<br>Sham: -13.5%
**Other pain syndromes**										
Sakrajai et al., 2014<br><br>Myofascial pain syndrome	Parallel-group RCT	Total: **31**<br><br>Active: 16<br>Sham: 15	1 mA<br><br>35cm²<br><br>*J* = 0.029	20 min	anodal tDCS over M1 (contralateral to the most painful side)	Total: **5**<br><br>Daily for 5 days	Follow-up<br>After 1, 2, 3 and 4 wks	NRS pain<br>Passive range of motion (PROM)<br>Analgesic medication use<br>WHOQOL – Physical functioning	tDCS combined with standard treatment<br>appears to decrease pain intensity.<br>Low benefit at the end of sessions and no benefit at follow-up	Active: - 40.3%<br>Sham: -13.5%
Hagenacker et al., 2014<br><br>Trigeminal neuralgia	RCT double-blind cross-over<br>design	Total: **10**	1.0mA<br><br>Anode: 16cm²<br>*J* = 0.062 mA/cm2<br><br>Ref elct: 50cm²<br>*J* = 0.012 mA/cm2	20’	Anode over M1 (no side specified)<br>Ref elct: SO contralal.	Total: **14**<br><br>daily for 2 wks	Before treatm.<br>After treatm.	Verbal Rating Scale (VRS) pain<br>Eletrophysiological:<br>- pain-related evoked potentials (PREP)<br>- nociceptive blink reflex (nBR)	Anodal tDCS reduced pain intensity significantly after two weeks of treatment. The attack frequency reduction was not significant. PREP showed an increased N2 latency and decreased peak-to-peak amplitude after<br>anodal tDCS. No severe adverse events were reported.	Active: -18.0%<br>Sham: +8.3%

Ibrahim et al., 2018<br><br>Visceral pain due to Hepatocellular carcinoma (HCC)	randomized, sham-controlled, double-blind, prospective study.	Total: **40**<br><br>Active: 20<br>Sham: 20	2 mA<br><br>35cm²<br><br>*J* = 0.057	30’	Anodal over M1 contral. Most painful area<br><br>Cathode over SO contral.	Total: **10**<br><br>Daily for 10 days	Before stimulation<br>After 1 day<br>After 5 days<br>After 10 days<br>After 1 month	Visual descriptor scale and VAS pain<br>HAMD<br>Side effects	VAS and VDS after the 10th session and one month later. Secondary outcomes were depression reduction on HAM-D.	Active: -46.1%<br>Sham: -21.6%
Lagueux et al., 2018<br><br>Complex regional pain syndrome	randomized parallel single blind	Total: **22**<br><br>Graded motor imagery (GMI) intervention + Active: 11<br><br>GMI + sham: 11	2 mA<br><br>5X7 cm<br><br>*J* = 0.057	20’	Anode over M1 contralat. to affected limb<br>Cathode over SO contralat.	Total: **14**<br><br>5 sessions/day for 2 wks and 1 session/day for 4 wks, all of them during GMI	Baseline<br>After 6 wks of treatment<br>1 month after the end of treatment	BPI-short form<br>SF-12<br>Tampa Scale of kinesiophobia (TSK)<br>PCS<br>STAI<br>BDI-II<br>Measures of compliance, adverse effects and blinding	GMI + tDCS induced no statistically significant reduction in pain compared to GMI + sham tDCS. Significant group differences in kinesiophobia, pain catastrophizing and anxiety at T1, but not maintained at T2	Active: -12.5%<br>Sham: -14.5%
Luedtke et al., 2015<br><br>Chronic low back pain	sham controlled double blinded randomised controlled trial	Total: **135**<br><br>Active: 67<br>Sham: 68	2mA<br><br>35cm²<br><br>*J* = 0.057	20’	Anode over left M1<br><br>Cathode SO contralat.	Total: **5**<br><br>Daily for 5 days before CBT	Before treatm.<br>After treatm.<br>After 4 wks<br>After 12 wks<br>After 24 wks	VAS (pain)<br>Oswestry disability index<br>Hannover functional ability questionnaire<br>Bothersomeness<br>RAND 36-item health survey<br>Fear avoidance beliefs questionnaire<br>Hospital anxiety and depression score<br>Patient perceived satisfactory	tDCS was ineffective for reduction of pain and disability and did not influence the outcome of cognitive behavioural management	Active: -12.5%<br>Sham: -14.6%

								improvement		
Oliveira et al., 2015<br><br>chronic temporomandibular disorders	Double-blinded parallel randomised clinical trial	Total: **32**<br><br>Active + exercises: 16<br>Sham + exercise: 16	2 mA<br><br>No info of elctr. size	20’	Anode M1 contral. to pain<br><br>Catchode So contralat.	Total: **5**<br><br>Daily for 5 days after exercises for cervical and mandibular areas	Before treatm.<br>After each day of treatm.<br>After 2 wks<br>After 3 wks<br>After 4 wks	VAS pain<br>severity of Temporomandibular disorder questionnaire (da Fonseca)<br>WHOQOL-BREF<br>Pressure pain threshold with algometer	Groups showed a decrease in pain intensity scores and increase in pressure pain threshold and quality of life, although there were no differences between the groups, indicating no additive effect of tDCS	Active: -49.1%<br>Sham: -42.8%
Silva-Filho et al., 2018<br><br>Chikungunya arthralgia	Parallel, sham, randomized, double-blind	Total: **19**<br><br>Active: 9<br>Sham: 10	2mA<br><br>35cm²<br><br>J = 0.057	20’	Anode left M1<br><br>Cathode SO contralat.	Total: **5**<br><br>Daily for 5 days	Before treatm.<br>After 1 day<br>After 5 days<br>After 1 wk	VAS pain<br>MPQ<br>BPI (short form)<br>Hand grip test (with dynamometer)<br>Upper and lower limbs flexion strength tests<br>SF-36	Significant reduction of quality of pain and pain felt on average and at the moment of assessment (post-treatment), as well as reduction of the impact of pain in general activities and normal work	Active: -74.5%<br>Sham: -39.3%
Morin et al., 2017<br><br>Provoked vestibulodynia	RCT parallel	Total: **39**<br><br>Active: 19<br>Sham: 20	2mA<br><br>35² cm<br><br>*J* = 0.057	20’	Anode M1 (no side specified)<br><br>Cathode SO contralat.	Total: **10**<br><br>Consecutive sessions daily, for 2 wks	Before treatment<br>After treatment<br>After 2 wks	Verbal numeric scale – pain<br>MPQ<br>Female sexual function index<br>Female sexual distress scale<br>Global measure of sexual satisfaction<br>patient treatment satisfaction and impression of change<br>Vaginal penetration cognition	No effects of tDCS in reducing pain during intercourse, vestibular sensitivity, or psychological distress, and to improve sexual function	Anodal: -16.8%<br>Sham: -24.0%

								questionnaire<br>PCS<br>IDATE<br>Pain anxiety symptoms scale<br>BDI		
Antal et al., 2010<br><br>Various syndromes	RTC crossover	Total: **12**	1mA<br><br>Anode: 4 x 4 cm<br>*J* = 0.062<br><br>Cathode: 5 x 10cm<br>*J* = 0.02	20’	Anode M1 left<br>Cathode SO contralat.	Total: **5**<br><br>Daily for 5 days	Before treatm. (between 10 days)<br>After each day of treatm.<br>After 1 wk<br>After 2 wks<br>After 3 wks<br>After 4 wks	VAS pain<br>Transcranial magnetic stimulation – cortical excitability<br>Adverse effects	Anodal tDCS led to a greater improvement in VAS ratings than sham<br>tDCS, evident even three to four weeks post-treatment.<br>Decreased intracortical inhibition was demonstrated after anodal stimulation.	Anodal: -35%<br>Sham: -1%

Notes. RCT = randomized clinical trial; *J* = current density; M1 = primary motor area; DLPFC = dorsolateral prefrontal cortex; SO = supraorbital region; Oz = occipital region; Cz = vertex region; VAS = visual analogue scale; NRS = numeric rating scale; FIQ = Fibromyalgia Impact Questionnaire; HADS = Hospital Anxiety and Depression Scale; SC-90R = Symptom Checklist - 90 – Revised; SF-26 = Medical Outcomes Study 36 - Item Short - Form Health Survey; LANSS = The Leeds Assessment of Neuropathic Symptoms and Signs; BDI = Beck Depression Inventory; PCS = Pain Catastrophizing Scale; STAI = State-Trait Anxiety Inventory; GDS = Geriatric Depression Scale; MMSE = Mini-mental State Exam; BPI = Brief Pain Inventory; QST = Quantitative Sensory Testing; WHOQOL = World Health Organization Quality Of Life instrument; HAMD = Hamilton Depression Rating Scale; MPQ = McGill Pain Questionnaire.
